# GPU accelerated digital twins of the human heart open new routes for cardiovascular research

**DOI:** 10.1038/s41598-023-34098-8

**Published:** 2023-05-22

**Authors:** Francesco Viola, Giulio Del Corso, Ruggero De Paulis, Roberto Verzicco

**Affiliations:** 1 Gran Sasso Science Institute (GSSI), L’Aquila, Italy; 2 European Hospital, Rome, Italy; 3 UniCamillus International University of Health Sciences, Rome, Italy; 4 University of Rome Tor Vergata, Rome, Italy; 5 POF Group, University of Twente, Enschede, The Netherlands; 6 Present Address: Institute of Information Science and Technologies A. Faedo, CNR, Pisa, Italy; 7 INFN-Laboratori Nazionali del Gran Sasso, Assergi (AQ), Italy

**Keywords:** Cardiology, Engineering

## Abstract

The recruitment of patients for rare or complex cardiovascular diseases is a bottleneck for clinical trials and digital twins of the human heart have recently been proposed as a viable alternative. In this paper we present an unprecedented cardiovascular computer model which, relying on the latest GPU-acceleration technologies, replicates the full multi-physics dynamics of the human heart within a few hours per heartbeat. This opens the way to extensive simulation campaigns to study the response of synthetic cohorts of patients to cardiovascular disorders, novel prosthetic devices or surgical procedures. As a proof-of-concept we show the results obtained for left bundle branch block disorder and the subsequent cardiac resynchronization obtained by pacemaker implantation. The in-silico results closely match those obtained in clinical practice, confirming the reliability of the method. This innovative approach makes possible a systematic use of digital twins in cardiovascular research, thus reducing the need of real patients with their economical and ethical implications. This study is a major step towards in-silico clinical trials in the era of digital medicine.

## Introduction

After the initial phase of research and development, the standard route for the transfer of a novel treatment to clinical practice is through randomised trials. In fact, every human is one of a kind and the efficacy of a new therapy can be assessed only via statistical analyses on large cohorts of patients. These are collected into randomised homogeneous groups and subjected to different treatments to compare the outcome of the new therapy with the established ones. However, recruiting enough participants for trials on rare or complex diseases could be very challenging while biased and incomplete cohorts yield inconclusive or misleading results. Paradoxically, clinical trials can thus become a barrier preventing some patients from accessing innovative treatments (not to mention the ethical question associated with sub-optimal or placebo therapies applied to some trial control groups). The generation of synthetic data by high-fidelity computer models might be an effective strategy to mitigate the above issues and this is one of the main aims of digital medicine. In fact, these models are referred to as digital twins and, when provided with appropriate input parameters, they can be used to surrogate real patients with ‘on demand’ features. In this way, the completion of thorough and cost effective clinical trials could be possible even in those cases in which enrolling a patients cohort would be challenging. The advantages of digital twins are huge since not only they can produce specific data but, in principle, they can anticipate the outcome of a surgical procedure, the progression of a disease or the performance of an implanted device thus shifting the medical paradigm from decisions based on past experience to predictions guided by virtual models.

Considerable efforts have been made in the last decade to produce digital twins for clinical applications and cardiac modelling has been among the fastest growing fields. The electrophysiology models and their numerical solution are nowadays well-assessed in the literature^1–3^, as well as their coupling with a structural solver yielding electromechanical systems^4–13^ enabling to reproduce the myocytes depolarization over the cardiac tissue including pathologic cardiac phenomena as ischemic events and defibrillation^14,15^. The influence of cardiac contraction on the electrocardiogram (ECG)^16^ and of the heart rate variability^17^ has also been included and recent review papers^18,19^ give a detailed account of whole-heart electromechanical models. Models including the hemodynamics are more scarce and the flow is often parametrised by simplified laws, as by considering only the blood pressure within each heart chamber while all valves are reduced to viscous resistances^20,21^ or by introducing a more realistic hemodynamics within a bi-ventricular configuration with simple lumped models for the heart valves^22,23^. More recently, an accurate model, which includes the atria and the hemodynamics, has been proposed^24^, although only the systolic function is considered and, therefore, the sealed atrio-ventricular valves are modelled as impermeable plane disks while the fully open semilunar ones as circular holes. Some heart models simultaneously accounting for the electromechanics and hemodynamics equations are currently being developed^23–26^, although they are generally very advanced for the electrophysiology and the elastodynamics of the tissues while the fluid dynamics, with its unsteady and transitional evolution, is often simplified. In some cases, the fluid motion in the left heart is solved using Navier-Stokes equations with kinematic driven^27^ and FSI^28^, whereas Navier-Stokes/LES equations have also been applied to reduce the computational burden^29^. From the above literature review, it appears that implementing a truly digital twin for the whole heart, capable of simulating all the features throughout the heartbeat is a formidable task which has not been fully accomplished yet. Furthermore, in order for a digital twin to be reliable and predictive, it must reproduce all the relevant dynamical details of the real counterpart thus requiring hundreds of million degrees of freedom. Even on modern supercomputers, such models entail simulation times of weeks or months and this prevents their routine clinical use: overcoming such limitation has huge cardiovascular potential and this has motivated the present work.

In this paper we present a groundbreaking virtual heart model coping with all the main features of the cardiovascular function: it accounts for the dynamics of the complex biological tissues both, active myocardium and passive valves, the transitional and turbulent hemodynamics, the myocardium electrophysiology and their strongly coupled interactions. The complete computer model uses up to one billion of spatial degrees of freedom and half a million time steps per heartbeat to capture with uncompromised accuracy the complex heart dynamics. The resulting huge computational burden is tackled by the latest graphics processing units (GPU) technologies which reduce the time-to-solution from months to a few days^30^. In the following we show first some results for a healthy heart with a physiological function then, by disconnecting the electrical conduction between the atrio-ventricular node and the left bundled branch, we induce its block and observe a deterioration of several cardiovascular indicators similarly to the clinical experience. Starting from this impaired configuration, cardiac resynchronization is simulated by pacemaker therapy and a small clinical trial is generated by varying the position of the implanted lead within the left ventricle. The outcome of the various virtual treatments is discussed in the light of the clinical experience (of one of the authors) and perspectives for future work are finally given.

## Results

**Figure 1:**
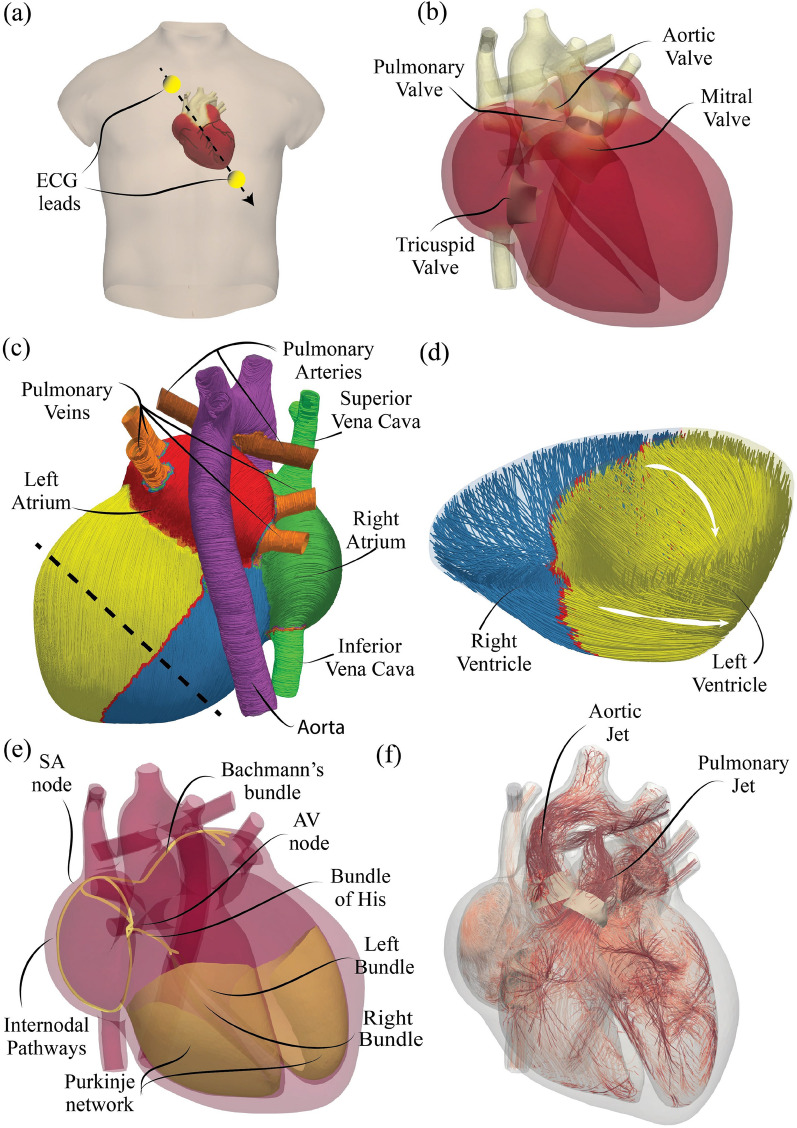
Geometrical and topological features of the cardiac digital twin. (**a**) Location of the heart model in a human torso and position of two virtual leads with which the ECG is computed (see section "Methods"). (**b**) Geometrical assembly of the heart model with the main elements, including veins and arteries. (**c**) Zonal separation of the heart with the external fibers orientation; the black dashed line is the trace of the cutting plane of panel **d**. The active and passive mechanical properties of the tissues are specific of each heart structure. (**d**) Plane section through the apical region of the ventricles to show the fibers orientation across the myocardium thickness. Note that the active contraction of the myocardium occurs along these directions thus yielding anisotropic and inhomogeneous features. (**e**) Hierarchical structures of the electrophysiological system: the conduction velocity of the electrical signal is position dependent. (**f**) Instantaneous snapshot of the flow streamlines at systole coloured with the velocity magnitude (0 m/s white, 1.5 m/s dark red); the clustering of lines in the ventricles evidences a swirling motion while the dark regions in veins and arteries show intense flows. The diastolic phase along with the corresponding snapshots in diseased and resynchronized conditions are reported in the supplementary material Fig. S1.

The quantities of interest, used to monitor the heart function and their dynamics, are obtained by our computer model whose details are given in the section "Methods". Here we add that the heart, including the four cardiac valves and main arteries/vessels, is properly located in a human torso (see Fig. 1a–d) and, during the simulations the electrical signals reaching the skin surface are detected to produce synthetic ECGs. In fact, in addition to the composite heart elastomechanics and hemodynamics^28^, the model accounts also for the complex, hierarchical structure of the electrophysiological system^31^, therefore it produces a realistic source of electric potential which propagates throughout the body.

**Table 1:** Normal ranges of cardiac parameters in healthy adults calculated as the mean value ± twice the standard deviation and corresponding parameters of the digital twin (healthy electrophysiology case).

Parameter	Source	Population (female)	Normal range	Digital heart
LV end diastolic volume (ml)	^33^	800 (462)	75–211	176
LV end systolic volume (ml)	^33^	800 (462)	24–92	86
LV stroke volume (ml)	^33^	800 (462)	45–125	90
LV ejection fraction (%)	^33^	800 (462)	48–72	51
RV end diastolic volume (ml)	^33^	800 (462)	74–234	189
RV end systolic volume (ml)	^33^	800 (462)	21–117	98
RV stroke volume (ml)	^33^	800 (462)	45–125	91
RV ejection fraction (%)	^33^	800 (462)	44–68	48
LA max volume (ml)	^33^	795 (462)	28–104	75
LA stroke volume (ml)	^33^	795 (432)	18–62	38
LA ejection fraction (%)	^33^	795 (432)	46–74	51
RA max volume (ml)	^33^	795 (432)	30–130	84
RA stroke volume (ml)	^33^	795 (432)	9–61	35
RA ejection fraction (%)	^33^	795 (432)	24–64	42
LV long axis diastole (mm)	^34^	52 (26)	62-98	92
LV short axis diastole (mm)	^34^	52 (26)	36-48	50
LV sphericity index diastole	^34^	52 (26)	0.40–0.64	0.54
RV long axis diastole (mm)	^35^	41 (21)	71.0–81.0	79
RV short axis diastole (mm)	^35^	41 (21)	27.0–33.0	32
Aortic annulus diameter (mm)	^36^	3370 (1156)	17.4–27.1	23
Pulmonary annulus diameter (mm)	^36^	3997 (1408)	19.5–30.8	22
Mitral annulus area (mm$^2$)	^37^	211 (114)	460–1220	800
Tricuspid annulus area (mm$^2$)	^38^	209 (116)	460–1260	800

Owing to the inherent human variability, defining a representative geometry of a heart is a problem in itself and two opposite directions can be taken: (i) replicating the heart of a particular individual (patient-specific model) or (ii) modelling a ‘normal’ organ with average properties. In this case the latter approach was pursued with the shape of each chamber, the local thickness of the tissues and their fiber directions obtained by surgical atlases^32^ or measurements whose ranges are reported in Table 1. It is worth mentioning that the heart resulting from these parameters does not belong to any specific individual but it rather exemplifies a standard configuration representative of the heart of adult humans. A typical run consists of a couple of initial heartbeats, during which the transient is accommodated, followed by ten cycles which are used to extract phase averaged quantities and statistics. The wall-clock time needed to solve a single beat of the whole cardiac dynamics strongly depends on the available hardware resources. For the fluid Eulerian grid at use (211′752′711 grid points) scaling tests have been run using both Nvidia V100 devices (on Marconi100, GPU cluster by Cineca) and the next generation Nvidia A100 devices on DGX machine. The wall clock time needed to solve a single time step is of 0.235s on 4 × V100 (a single Marconi100 node), which reduces to 0.1285s and 0.0814s using 4 × and 8 × A100 devices, respectively. In the last case, $\approx 11.3$ h are needed to solve a single heartbeat and to produce a database of $\approx 8$ Tbytes to be analysed by successive postprocessing.

### Physiological conditions

**Figure 2:**
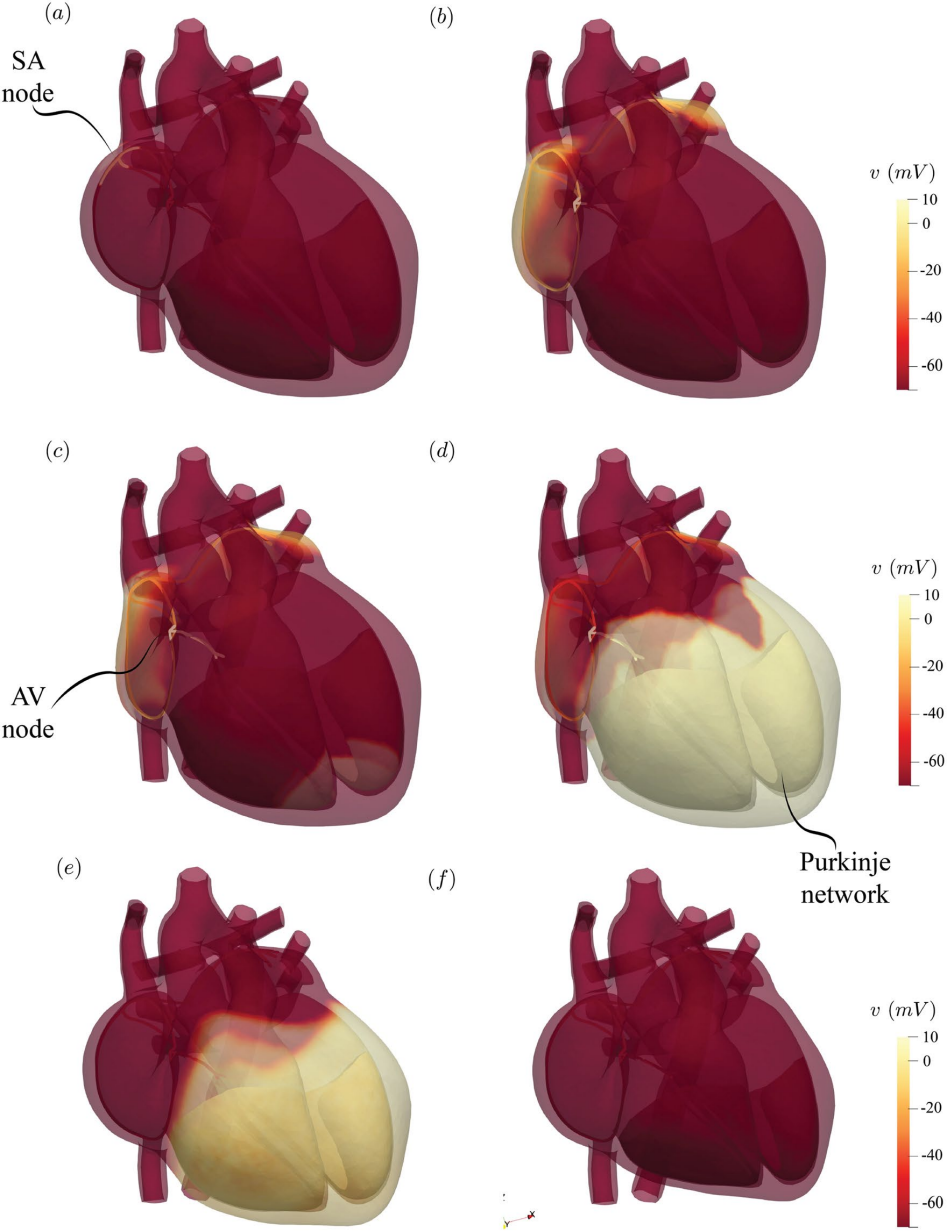
Depolarization of the electrophysiology network. Instantaneous snapshots of the activation potential during the heartbeat: (**a**) The sino-atrial node ‘sparks’ the initial triggering signal ($t\simeq 0$ s); (**b**) The electrical signal spreads quickly, via the internodal pathways, across the atrial tissue and depolarises them ($t= 160$ ms); (**c**) The signal reaches the atrio-ventricular node where it is delayed by $\approx 100$ ms by the very small conduction velocity of the signal in that region ($t= 190$ ms); (**d**) At $t=250$ms, the activation potential has spread through the bundle of His, the Purkinje fibers and the myocardial tissue of the ventricles; (**e**) While the myocardium repolarizes a vigorous contraction starts ($t=400$ ms); (**f**) The ventricles attain the strongest contraction at $t=560$ ms, a long relaxation period follows until the beginning of the next heartbeat.

**Figure 3:**
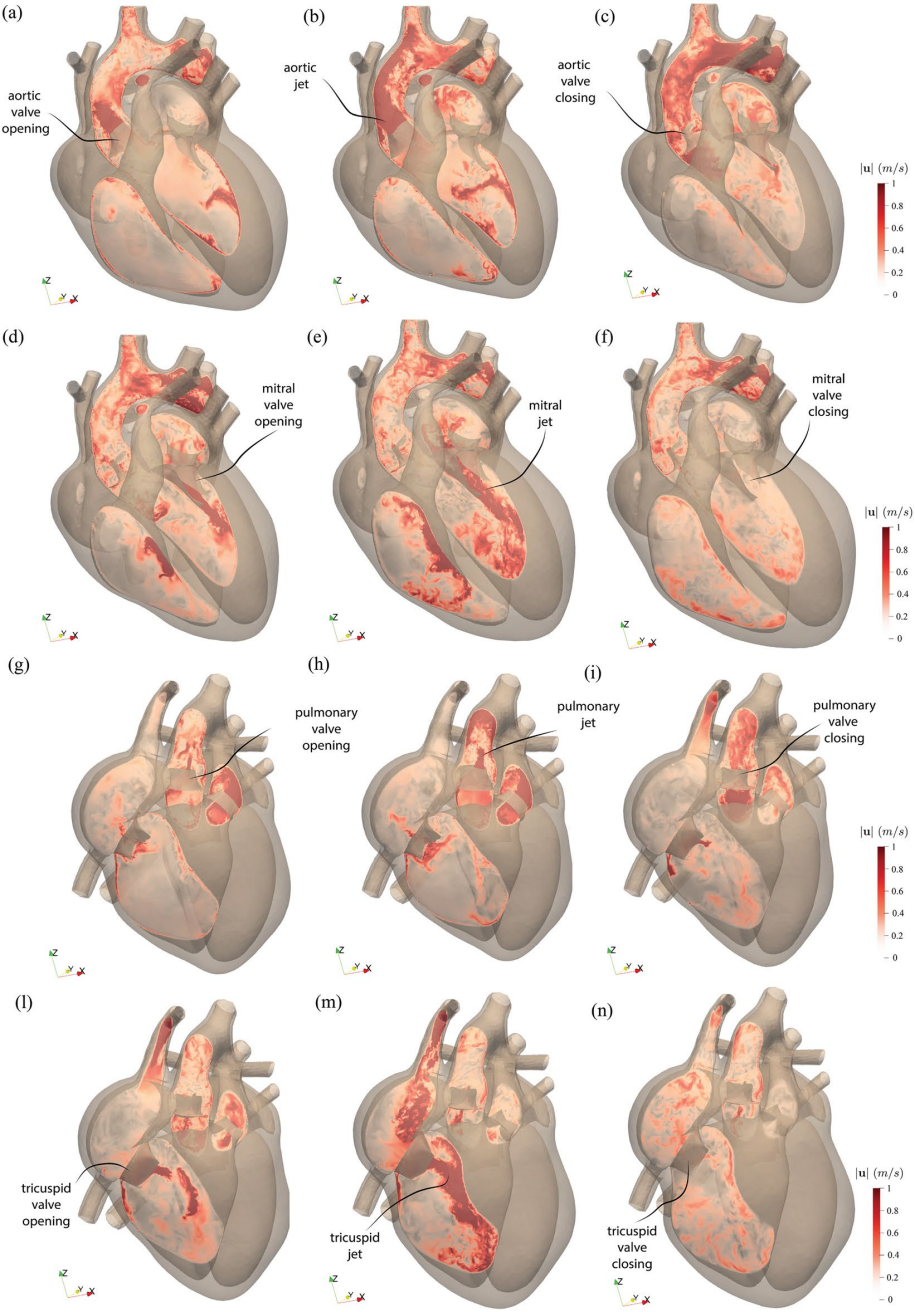
Cardiac hemodynamics. Instantaneous snapshots of the blood velocity magnitude over plane sections crossing the left (**a**)–(**f**) and right (**g**)–(**l**) parts of the heart. The left plane position is such to cross in the middle the mitral and aortic valves. Similarly, the right plane crosses the pulmonary and tricuspid valves. (**a**) and (**g** $t=500$ ms, (**b**) and (**h**) $t=540$ ms, (**c**) and (**i**) $t=600$ ms, (**d**) and (**j**) $t=620$ ms, (**e**) and (**k**) $t=680$ ms, (**f**) and (**l**) $t=780$ ms.

**Figure 4:**
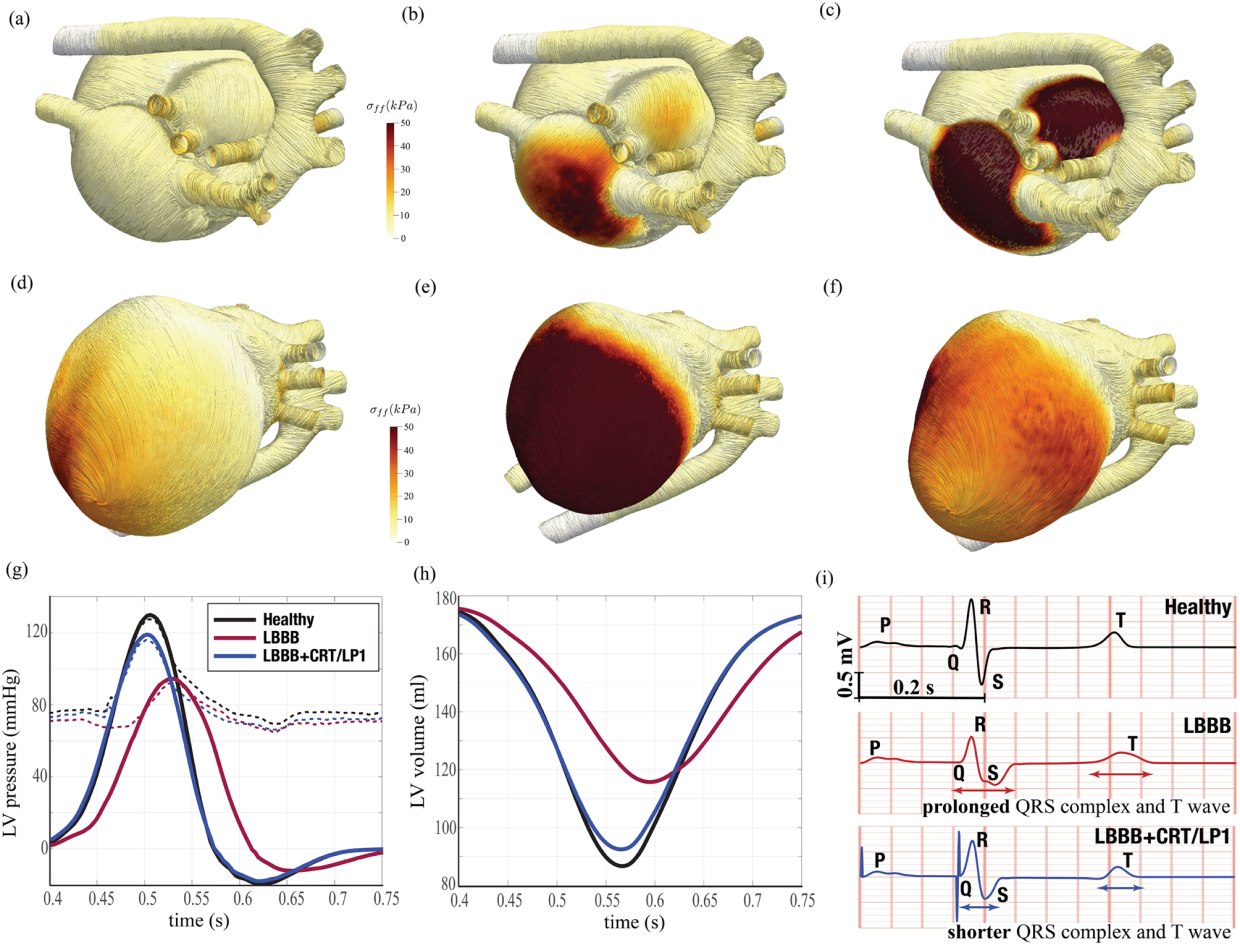
Tissue stresses and clinical indicators. (**a**)–(**f**) Instantaneous surface distribution of the tension along the fibers axes (force per unit area) during a heartbeat; during diastole, when atria contract, the heart is viewed from above (panels (**a**)–(**c**), during systole (**d**)–(**f**) the viewpoint is from below to evidence ventricles contraction. **a,** $t=20$ ms, (**b**) $t=120$ ms, (**c**) $t=260$ ms, (**d**) $t=460~$ms, (**e**) $t=560$ ms, (**f**) $t=640$ ms. (**g**) Time evolution of the left ventricle blood pressure during systole: black solid line for a healthy heart; red solid line for the impaired heart with a left bundle branch block (LBBB); blue solid line for the impaired heart after resynchronization (CRT, with left ventricular lead in the position LP1). The dashed lines have the same meaning as before but for the aortic pressure. **h,** Time evolution of the left ventricle volume during systole, the colour code is the same as in panel (**g**). (**i**) ECG trace from the two sensors as in Fig. 1a), the colour code is the same as in panel (**g**).

The reference healthy case is generated by running the model under nominal conditions and some representative results are given in Figs. 2, 3, 4, respectively for the electrophysiology, hemodynamics and the tissue mechanics.

**Figure 5:**
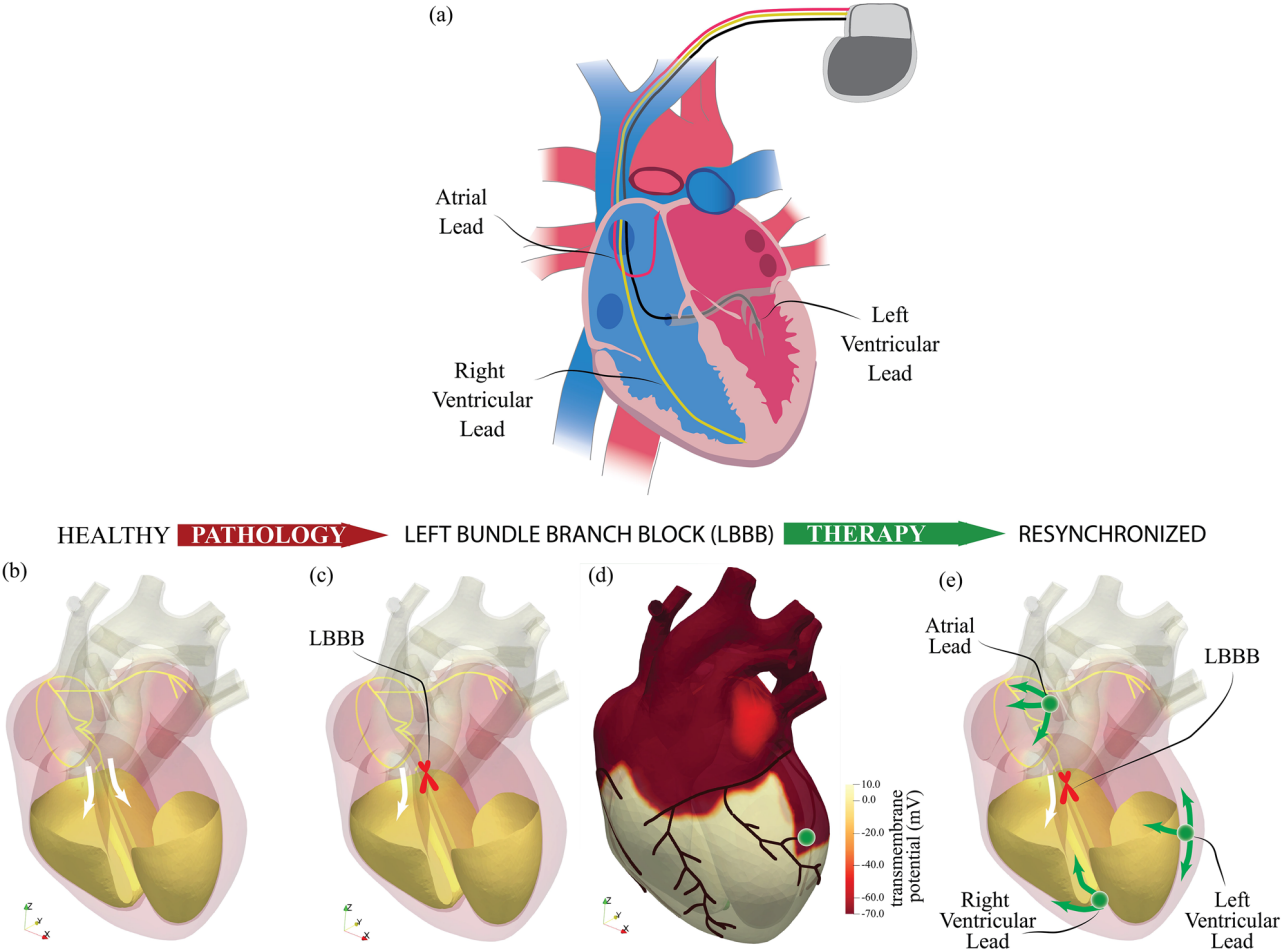
Cardiac resynchronization therapy. (**a**) Sketch of the biventricular pacemaker device with the atrial lead (in red), right ventricular lead (in yellow) and left ventricular lead (in green). (**b**) Arrangement of the fast conducting structures of the electrical signal in the heart. The two white arrows evidence the branching of the signal in the Bundle of His, after the Atrio-Ventricular node. (**c**) The same as (**b**) but with a red cross indicating the point where the electrical connection for the left side has been interrupted. (**d**) Surface distribution of the activation potential in the myocardium (at $t=316$ ms) for the configuration in panel (**c**) with overlapped the position of the main veins; the green bullet indicates the optimal point for the lead implantation as it can be reached via the coronary vein and is located within the polarised portion of the tissue. (**e**) Same configuration as in panel (**c**) with the position of the three pacemaker leads. Note that in this configuration the atrial and right ventricle leads operate only as sensors since only the left ventricle lead is allowed to issue triggering signals.

Figure 2 shows the depolarization pattern which starts from the sino-atrial node and quickly proceeds through the atria via the fast conducting bundles. The signal then slows down in the atrio-ventricular node for about 100 ms to allow the fully contracted atria to complete the filling of the relaxed ventricles. A quick propagation follows along the His bundle and the Purkinje network to depolarize the ventricles and lead to their strong (almost) synchronous contraction (see also Fig. 5b) . The electrically driven contraction and relaxation of the tissues squeezes the blood from atria to ventricles and then to veins and arteries following precise directions which are ensured by the passive opening and closing of the heart valves. Figure 3 shows the flow structure during several instants of the heartbeat and, since a single planar section cannot describe the complex structure of the heart, the flow on two different planes for the left and right heart is shown. Furthermore, the supplementary material Fig. S1a,b report three-dimensional visualizations of the systolic and diastolic hemodynamics through instantaneous streamlines of the blood velocity.

For the sake of completeness, in Fig. 4, the intensity of the tissue contraction is visualised through tension stress along the tissue fibers with results which are complementary to the activation potential of Fig. 2 and the produced hemodynamics of Fig. 3.

Although a high-fidelity digital model makes easily accessible the complex three-dimensional dynamics of the various heart systems the same is not true in the routine clinical practice which, instead, relies on simpler quantities that can be directly measured or inferred through standard analyses. Examples are the pressure variations during a heartbeat, the volume of the left ventricle and the ejection fraction or the ECG, as shown at the bottom of Fig. 4. The values obtained for the healthy configuration are 130/76 mmHg for systolic/diastolic pressure, $\approx 51\%$ for the ejection fraction and an ECG trace showing the appropriate duration of the QRS complex and T wave.

We wish to stress that all these quantities have been obtained as part of the model results without additional inputs other than the electro-mechanical properties of the system thus providing evidence of its predictive capability.

### Pathological left bundle branch block

A further step forward for the model assessment is to show that not only it behaves correctly in healthy physiological cases (for which it has been designed) but it also reproduces the pathologic conditions of a specific induced disfunction. In order to accomplish this goal, we have disconnected the electrical conduction between the atrio-ventricular node and the left His bundle (Fig. 5c) thus causing a left bundle branch block (LBBB) disorder.

**Figure 6:**
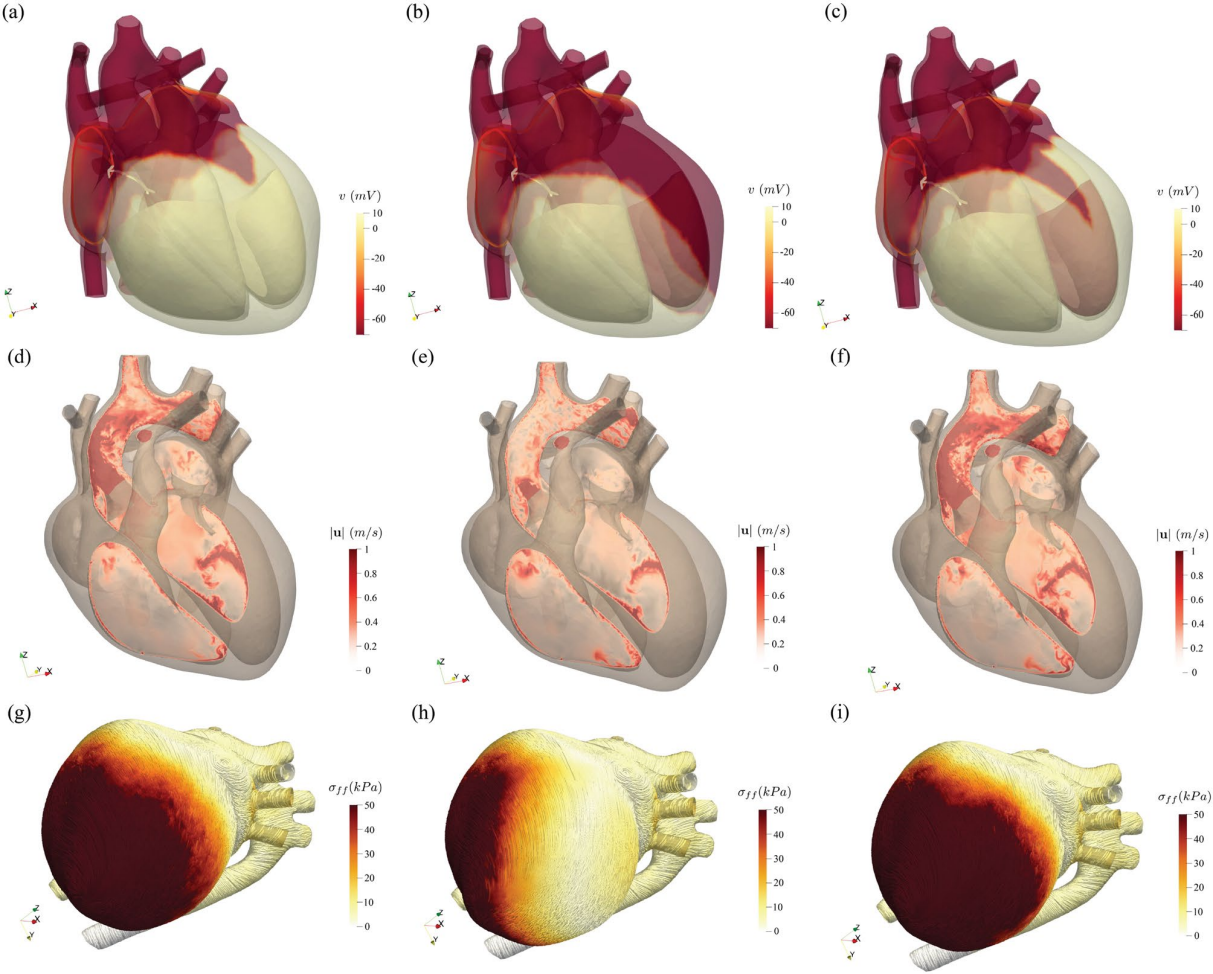
Pathological effect and therapeutic effectiveness. Comparison of different quantities for healthy (**a**), (**d**), (**g**), impaired (**b**), (**e**), (**h**) and resynchronized (**c**, **f**, **i**) heart during systole. (**a**)–(**c**) Instantaneous surface distribution of the activation potential ($t=252$ ms). (**d**)–(**f**) Blood velocity distribution on a planar section cutting the left heart at peak systole ($t=520$ ms). (**g**)–(**i**) Surface distribution of the tension along the fibers axes at peak systole (force per unit area, $t=520$ ms).

The immediate consequence is that the tissue depolarization proceeds quickly in the right ventricle along the Purkinje fibers but it is much delayed on the left counterpart as the activation potential can propagate only through the slow conducting myocardium: this is evident in Fig. 6b showing the largely polarized (not contracted) posterior region of the left ventricle compared with the fully contracted myocardium of the healthy case (Fig. 6a,g; see also Fig. S4 in the supplementary material). The hemodynamics produced by the impaired left ventricle function yields a weak aortic jet evidenced by Fig. 6e and in the supplementary material Fig. S2. Also the myocardium contraction is consistent with the above picture and Fig. 6h (see also supplementary material Fig. S11) confirms that the left ventricle fails to reach the same contraction strength as the right part.

Concerning the classical clinical indicators, we see that the peak left ventricle and aortic systolic pressures drop by about $30\%$ (95/69 mmHg) and the systole duration is extended in the cycle. The ejection fraction decreases to a value of $34\%$ with the ECG trace evidencing slower repolarization, prolonged QRS duration and QT interval which are all common indicators of the LBBB disorder.

### Effect of cardiac resynchronization therapy

Cardiac resynchronization therapy (CRT) is indicated in patients with heart failure evidenced by depressed ejection fraction and wide QRS complex in the ECG trace. In short, CRT consists of the implantation of a pacemaker which using artificial electrical signals restores the coordination of ventricles contraction. A common device is the biventricular pacemaker which has three leads implanted, respectively, in the upper part of the right atrium, in the apex of the right ventricle and in the posterior wall of the left ventricle. The leads are inserted via the upper vena cava and the left ventricle is reached passing through the coronary sinus; as a consequence, it can be implanted only in the regions crossed by its main tributary veins (Fig. 5a). On the other hand, the most appropriate positioning would be the latest depolarized point of the left ventricle whose position neither is known precisely nor is necessarily reached by a main vein. In Fig. 5d,e we show the optimum implantation point (hereafter indicated as LP1—*lead position one*), according to the above criteria, with the lead activation time tuned so to yield the maximum cardiac output. Figure  6 shows the activation potential, the hemodynamics and tissue contraction after the resynchronization therapy which exhibit features similar to the physiological case; see also supplementary material Figs. 3, 5 and 12 for the snapshots sequence during the heartbeat. Further quantitative confirmation comes from the standard clinical indicators of Fig. 4g–i whose values and time evolution closely match those of the healthy reference case. In particular blood pressure values recover to 120/72 mmHg while the ejection fraction raises to $48\%$ with the ECG trace which regains the physiological timings.

#### Sub-optimal left-ventricle lead implantation

For real patients, instantaneous maps of the activation potential such as that of Fig. 5d are not available and the exact location of the latest depolarised left-ventricle region is not known a-priori. Furthermore, the main myocardium veins form a very sparse network thus the left-ventricle lead is unlikely to be implanted in the best possible position and the initial sub-optimal outcome is usually improved by successive tuning of timings and delays among the atrial and ventricular leads.

**Figure 7:**
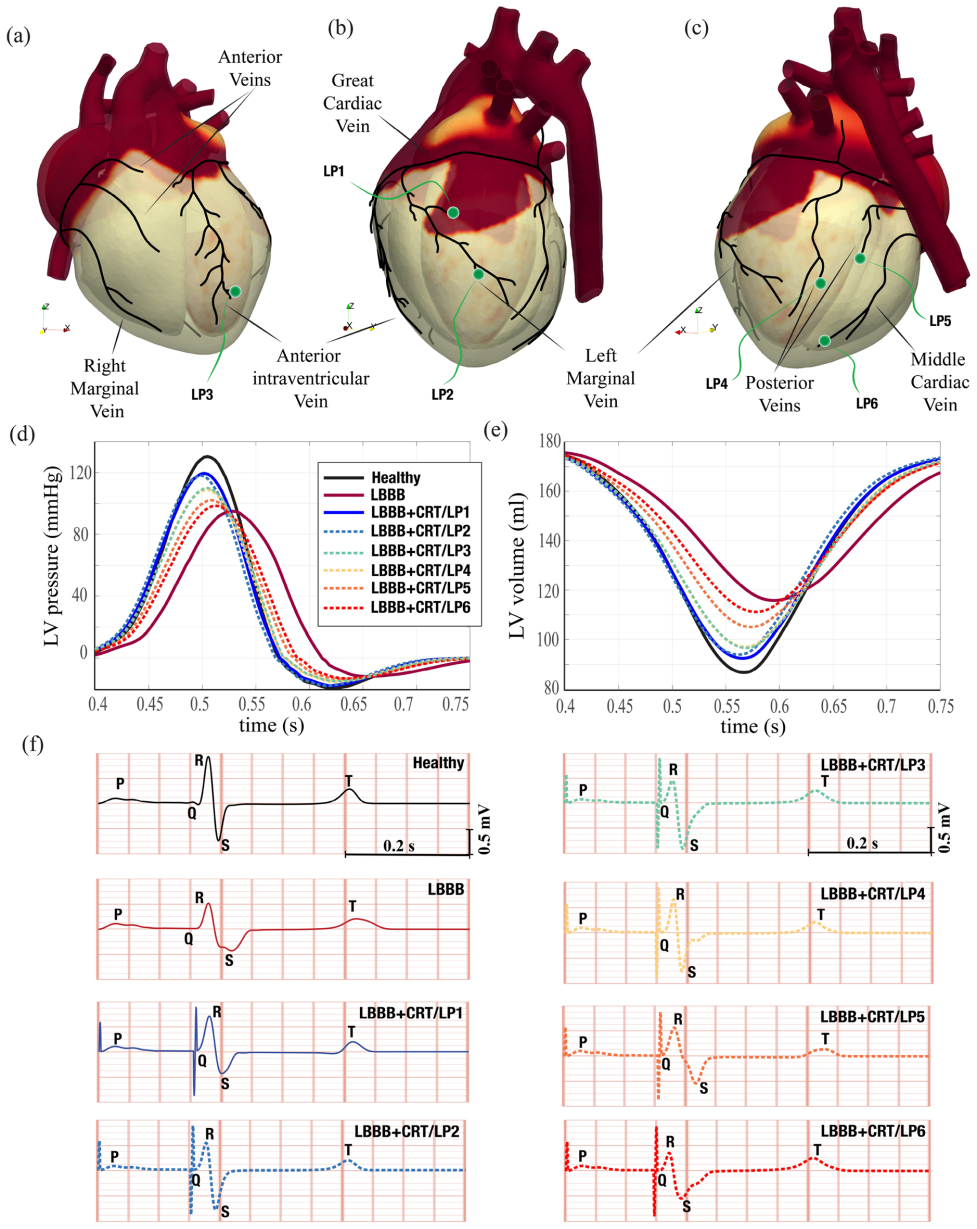
Optimal and suboptimal left ventricular pacing. (**a**)–(**c**) Views of the different possible positions for the left ventricular lead with a sketch of the main veins arrangement. **d,** Time evolution of the left ventricle blood pressure during systole: black solid line for healthy, red solid for impaired and blue solid for the heart after optimal resynchronization. The dashed lines represent the other resynchronization cases for different lead positions as detailed in panels (**a**)–(**c**). (**e**) Time evolution of the left ventricle volume during systole, the colour code is the same as in panel (**d**). (**f**) ECG trace from the two sensors as in Fig. 1a), the colour code and labels are the same as in previous panels.

Nevertheless, depending on the particular lead position, the cardiac function improves only up to a given threshold and in Fig. 7 we report the results of a simulation campaign, in which the left ventricle lead has been implanted in five possible alternative positions. For each case, denoted by LP2–6, the activation time of the left ventricle lead has been tuned, by complementary simulations, so to obtain the best cardiac output similarly to the procedure following real implantation surgery. The data are presented in the same form as for the previous cases and, in the sake of conciseness, the corresponding maps of activation potential blood flow and fibers tension distribution over the tissue are reported in the supplementary material Figs. S6–S10 and Figs. S13–S17. The emerging picture from the results of Fig. 7d–e is that the cardiac function improved in all cases although the recovery is the smaller the farther is the implantation point from the optimal position identified by the LP1 case. Similar indication comes from the ECG traces of Fig. 7f when comparing the duration of the QRS complex and the repolarization time delay with the values of the healthy reference case.

More quantitative data about the efficacy of the resynchronization procedure is summarized in Table 2 in which volumes, pressures and derived quantities are computed for all the simulated cases. As the position of the left ventricular lead is moved from the optimal position LP1 to the suboptimals ones LP2-6, the end systolic volume increases, thus corresponding to a decrease of the stroke volume, of the ejection fraction and of the peak systolic pressure.

## Discussion

In this paper we have presented a GPU-accelerated cardiac model for determining the changes produced by pathologies or the outcome of a therapy. The multi-physics solver encompasses (i) the complex dynamics of the cardiac tissues that are either passively moved by the interaction with the flow (valves and artery/vein walls) or actively deformed by the propagation of an electrical signal through the myocardium via the electrophysiology network of the heart (ii) the material properties of the various tissues that are anisotropic and have nonlinear constitutive relations, (iii) the pulsatile, transitional and turbulent character of the flow that requires the state-of-the-art direct numerical simulation for the correct description of all the flow scales up to the smallest.

As a proof-of-concept, for the use of a human heart digital twin to study specific features of the cardiac function, the model has reproduced the physiological behaviour when run in healthy conditions while pathological alterations have emerged after having induced a disorder.

Finally, the same model has predicted the outcome of a resynchronization treatment aimed at restoring the cardiac function; in order to account for the inherent uncertainties related to the clinical procedure, different positions of the left ventricle pacemaker lead have been tested and the results have yielded the whole range of possible outcomes, from full recovery to marginal improvements.

The complete set of simulations presented in this paper can therefore be regarded as a proof-of-concept for a small clinical trial aimed at assessing the effect of uncertainty in the positioning of the ventricular lead of a pacemaker device. In real clinical practice this would be achieved by collecting data from different patients and performing a retrospective statistical analysis. However, each patient is different from the others, therefore when comparing different outcomes it is practically impossible to separate the effects of the surgical procedure from the epistemic variability of each individual. In contrast, the present model produces different cases simply by changing one or more input parameters which, therefore, can be assigned to form a representative cohort of patients in clean, repeatable and controllable conditions.

In fact, clinical trials infer the quantities of interest (QoIs) by comparing the outcome of alternative treatments on different cohorts of homogeneous patients. These should include a number of individuals large enough to properly represent the statistics of the population in turn entailing a random sampling. This is equivalent to a Monte Carlo analysis (MC), which gauges the size *N* of the cohort needed to compute the statistics; since the error in estimating statistical moments^39^ decays as $\sim 1/\sqrt{N}$, a cohort of about $N=400$ patients is needed to reduce the uncertainty below $5\%$ while it ramps up to $N=10000$ for a threshold of $1\%$.

**Table 2:** Main cardiac parameters as obtained from the model for the various healthy, pathological (left bundle branch block, LBBB) and treated cases (with cardiac resynchronization therapy, CRT).

Parameter	Healthy	LBBB	LBBB+CRT LP1	LBBB+CRT LP2	LBBB+CRT LP3	LBBB+CRT LP4	LBBB+CRT LP5	LBBB+CRT LP6
LV end diastolic volume (ml)	176	176	176	176	176	176	176	176
LV end systolic volume (ml)	86	116	92	94	97	97	105	111
LV stroke volume (ml)	90	60	84	82	79	79	71	65
LV ejection fraction (%)	51	34	48	47	45	45	40	37
Max LV pressure (mmHg)	130	95	120	118	110	109	102	98

The labelling of the cases is the same as in Fig. 7.

When resorting to in-silico trials, however, the features of virtual patients can be defined on demand and this allows the use of more efficient sampling strategies which ensure a faster convergence than MC. For example, using a variance reduction technique (such as the Latin Hypercube sampling^40^), the error decreases as $\sim C/N^{1/2}$, with the constant $C \le 1$^39^. The converge of the QoIs statistic can be further improved considering the so called quasi-random sampling strategies, such as the Sobol’ low discrepancy sequence^41^. In this case the error decays as $1/N^\alpha$, with the exponent $\alpha$ in the range [1/2, 1]^42^. It appears that combining a quasi-random method with a moderate variance reduction technique entails a significant reduction of the number of samples and, consequently, the size of the virtual patients cohort. For example, with $\alpha = 0.7$ and $C=0.08$, an in-silico study would need $N=50$ ($N=500$) samples to estimate QoIs within $5\%$ ($1\%$), rather than $N=400$ ($N=10000$) of a standard MC method routinely employed in the clinical practice. The advantages associated with in-silico trials and the optimal sampling techniques are even larger when the analysis is focussed on rare diseases. In fact, estimating events with low probability ($p \ll 1$) yields a prohibitively slow convergence rate ($\sim 1/\sqrt{pN}$ for standard MC methods while, using a method like the Subset Simulation^43^, which sequentially samples the distribution tails, the error decays as $\sqrt{(\log (p^{-1})^2/N}$^44^. This implies that, for an uncertainty threshold of $\approx 10\%$, an event of probability $p=10^{-2}$ needs a cohort of 10000 patients for MC sampling and only about 400 with a Subset Simulation approach.

Cardiac digital models can thus be exploited to run in-silico clinical trials for investigating pathologies and for testing the outcome of surgical procedures or devices implantation. As an example, the effect of a myocardial or valvular pathology on the normal hemodynamics (and consequently on the wall shear stress, tissue damage and hemolysis) can be studied, and the beneficial effects of implanted cardiac device or prosthesis can be predicted and quantified numerically. Nevertheless, the high computational cost of the simulations calls for high-performance computing facilities to reduce the time-to-solution and an efficient code parallelization with the effective use of the computational resources is key, especially for running simulations campaigns. The GPU-accelerated multi-physics computational model proposed here allows to solve a heart beat in less than 12 h running on 8$\times$A100 devices, which corresponds to a single DGX node. Such wall clock time will be further reduced keeping up with the upcoming hardware improvements. As an example, a speed up of about two will be achieved with the next Nvidia H100 devices (which have been released in the first quarter of 2023) and more performant devices are expected in the next years. In principle, the time-to-solution can be also reduced by running a multi-node simulation, thus resorting to more GPU devices, even if the connection among nodes (Infiniband) is slower than the NVLink connection between the GPU devices within the same node. Consequently, as the simulation is scaled out from single node to larger node counts, even if the wall clock time reduces, an increasing percentage of the buffer is sent over the slower connections, which causes a reduction in performance. Therefore, in the perspective of running in-silicio clinical trials and minimize the total wall clock time for solving a patient cohort (rather than a single cardiac simulation) it is more efficient to solve one simulation per node in a GPU cluster, where each node is equipped with 4 to 8 devices having a total of 80 Gb RAM per device.

Before concluding this paper we would like to stress that despite the effort made to develop a complete model for the whole heart, still there are many limitations. In our model the nonlinear stiffening at high strains observed in the cardiac tissue is modeled suitably by a Fung-type constitutitve relation based on the exponential function for the strain energy function, whose parameters depend on the local muscular fiber, thus accounting for the orthotropic nature of the tissues. The constitutive modelling can be further improved for instance using the Holzapfel Ogden relation^45^, which has been demonstrated to capture better the passive mechanical behavior of arteries and 3-D states of deformation especially in the case of shear deformation. Another key challenge to be tackled in future studies is addressing the inherent human variability and the uncertainty of the heart tissue parameters, which could yield significant difference in the patient response to therapies and surgical procedures. In this scenario, a large simulation campaign is needed to determine the population response to CRT by sweeping the probability distributions of the uncertain inputs of the digital twin, such as the elastic parameters of the tissues, electrical conductivities, the orientation of the muscular fibers and the geometry of the heart chambers, just to mention a few.

Furthermore, the heart is not just a complex electomechanical system but it relies also on many biochemical processes which, at the moment, are not modelled by our digital twin. For example, coupling continuum mechanotransduction models can be very useful for predicting the evolution of abnormal haemodynamics or the progression of a disease diagnosed at the initial stage^46^. In this framework the endothelial wall shear stresses (WSS) are an important input parameter for these models, as abnormal WSS distributions may alter the physiological stress levels and activate tissue remodelling or enhance calcification^47^. The accurate solution of WSS calls for a local grid refinement at the wet tissues to solve the steep velocity gradients within the boundary layers. In immersed boundary methods (IBMs; also adopted here, see section "Methods"), as the grid directions are not aligned with the wet tissues which significantly deform and change orientation during a heartbeat, a fine grid size should be used in the three spatial directions throughout the whole computational domain^48^, thus increasing the computational burden. On the other hand, the remodelling processes, evolve over times which range from seconds to months and many effects tend to be cumulative triggering feedback loops whose result can show up in years. Such a long time scales are clearly out of reach of our computer model which, at most, could reproduce a few minutes of heartbeats. A possible strategy to bridge this gap could be to rely on continuum mechanostransduction models in which the results of high-fidelity hemodynamic simulations are used as input for biological tissue models which, in turn, can predict the long term evolution of a given situation. In case the evolution of the system produces a change of geometry (as for tissue remodelling), iterations between the digital twin of the heart and mechanotransduction models would be necessary in order to predict the final evolution of an initial symptom: the combination of such a sophisticated computer models is at the base of digital medicine and is one of the main challenges of future research.

## Methods

### Cardiac geometry

The 3D heart geometry including the four cardiac valves and the main vessels has been built using modeling softwares (*Rhinocad, Blender, MeshMixer, Meshlab*) so as to reproduce high-resolution clinical images and medical atlas, where the corresponding lengths and thicknesses are within the normal ranges reported in Table 1. In the left part of the heart, the left atrium (red chamber in Fig. 1c) receives oxygenated blood via the pulmonary veins (orange veins in the same figure) and is connected to the left ventricle through the mitral valve which has two leaflets, an anterior next to the aortic valve and the other posterior close to the lateral myocardium (see Fig. 1b). The left ventricle (yellow chamber) pumps blood through the aorta causing the three-leaflets aortic valve (see Fig. 1b) to open during systole and to close during diastole. On the other hand, the right atrium (green chamber in Fig. 1c) receives deoxygenated blood from the superior and inferior vena cava (green veins) and is connected to the right ventricle through the tricuspid valve that has three leaflets (see Fig. 1b). The right ventricle (blue chamber) pumps blood through the three-leaflets pulmonary valve (see Fig. 1b) towards the pulmonary artery (brown artery in Fig. 1c). The heart tissues are made of fibers which make their electrical conductivities and elastic properties orthotropic. In particular, the muscular fibers of the ventricular myocardium have a dual-orientation^49,50^, with directions ranging approximately from $+60^{\circ }$ to $-60^{\circ }$ across the ventricular wall^51^, whereas atrial fiber orientation is uniform within the myocardium thickness^52^, see Fig. 1c,d.

The Lagrangian mesh used for the structural and electrophysiology solver of the heart is described by $\sim 5\times 10^5$ cells including the four cardiac valves. The heart geometry is *immersed* in a computational box for the hemodynamics of $L_x\times L_y\times L_z = 10\times 10\times 14\ \mathrm{cm^3}$ that is discretized, by an Eulerian mesh of $531\times 531\times 751$ nodes corresponding to a grid spacing $\le 190$ μm, which is needed to correctly solve the hemodynamics. A small time step of about 2 μs is needed to advance a single heart beat, which corresponds to 500’000 time steps with a heart rate $\text {HR}=60$ bpm.

### Fluid-Structure-Electrophysiology interaction (FSEI)

The digital twin of the human heart is based on a multi-physics computational model tailored to accurately solve cardiovascular flows, which can cope with the electrophysiology of the myocardium, its active contraction and passive relaxation, the dynamics of the valves and the hemodynamics within the heart chambers and arteries. These models are three-way coupled with each other, thus capturing the fully synergistic physics of the heart functioning and the resulting FSEI is here summarized.

**Figure 8:**
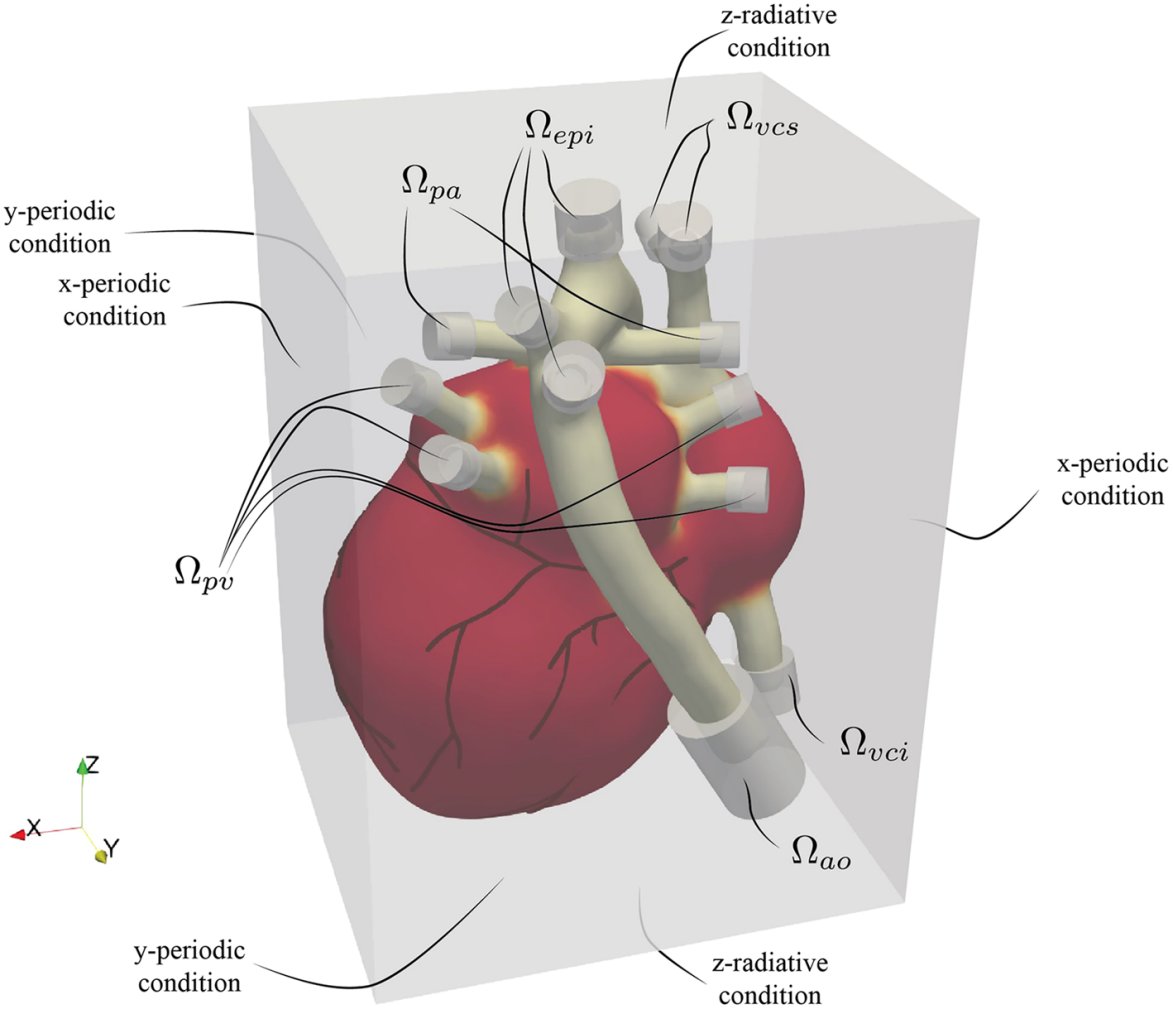
Boundary conditions of the cardiac model. The whole cardiac geometry is immersed in the computational domain of the blood phase (external bounding box). The grey volumes at the tip of the artery/veins indicate the region where the impedance of the missing circulation is mimicked by a volume forcing.

#### Fluid solver

The blood velocity ${\textbf{u}}$ and pressure *p* are governed by the incompressible Navier–Stokes and continuity equations:

1 $$\begin{aligned} \rho \left( \frac{\partial \textbf{u}}{\partial t} + \nabla \cdot ( \mathbf{u \textbf{u}}) \right) = - \nabla p + \nabla \cdot {{\varvec{\tau }}} +\mathbf{f_{IB}} +\mathbf{f_{WK}}, ~~~~ \nabla \cdot \textbf{u} = 0, \end{aligned}$$

where $\rho =1060~\text {Kg/m}^3$ is the blood density. In the case of a Newtonian fluid, the viscous stress tensor is given by ${\varvec{\tau }}= \mu (\nabla +\nabla ^T) {\textbf{u}}$ with $\mu =3.5~\text {mPa s}$ the fluid viscosity, whereas non-Newtonian fluids call for more complex constitutive relations. Blood is a concentrated suspension of cells, in a Newtonian liquid, the plasma, therefore its overall behaviour is that of a non-Newtonian fluid owing to the surface tension of the cell membranes on the Newtonian matrix. In order to account also for this behavior, a non-Newtonian (shear-thinning, Carreau-Yasuda^53^) blood model has been implemented in the flow solver even if it has been shown that the non-Newtonian blood features become relevant only in vessels of sub-millimeter diameter while in the ventricular flow they produce only minor effects. The governing equations (1) are solved over Cartesian meshes using central second-order finite-differences discretized on staggered grids, whereas the equations are marched in time using a fractional step with an explicit Adams–Bashforth method for the nonlinear convective term and an implicit Crank-Nicolson method for the viscous terms^28,30^.

As it happens in immersed boundary methods (IBMs), the heart is immersed in the fluid domain (Eulerian grid), as shown in Fig. 8. The no-slip condition on the moving wet heart tissues is imposed through the instantaneous forcing $\textbf{f}_{IB}$ using an IBM based on the moving least square (MLS) interpolation^54,55^, which is used to transfer the IB forcing computed at the Lagrangian markers (uniformly distributed on the wet surface of the IB^55^) to the Eulerian grid. An advantage of this technique with respect other direct IBMs^48^, is that the same MLS interpolation is also used to transfer back pressure and viscous stresses from the Eulerian grid to the Lagrangian markers, thus obtaining smooth hydrodynamics loads which are provided as input to the structural solver for fluid-structure coupling. In the case of the valve leaflets, both sides of the tissues are wet by the fluid and the local hydrodynamic force is computed over both the positive $\textbf{n}^+$ and negative $\textbf{n}^-=-\textbf{n}^+$ normal directions: $\textbf{F}_f^{ext} = [ -(p^+_f - p^-_f) \textbf{n}^+_f + {\varvec{(}} {\varvec{\tau }}^+_f -{\varvec{\tau }}^-_f) \cdot \textbf{n}^+_{f} ] A_f$, where $A_f$ is the area of the triangular face. On the other hand, for closed surfaces, like the heart chambers and vessels, hydrodynamic loads are only computed over the inner surface as: $\textbf{F}_f^{ext} = [ -p_f \textbf{n}_f + {\varvec{\tau }}\cdot \textbf{n}_f ] A_f$, being $\textbf{n}$ the wet normal direction. The hydrodynamic loads evaluated at the wet faces are then transferred to the wet nodes thus obtaining, $\textbf{F}_n^{ext}$, used in the Newton’s equation in the next paragraph.

As visible in Fig. 8, the tips of the arteries and veins representing the inlets/outlets of the heart do not cross the boundaries of the fluid computational domain, and during the cardiac dynamics blood is sucked from the outer volume through the pulmonary veins and superior/inferior vena cava and propelled towards the same outer volume through the aorta and the pulmonary arteries. However, the heart is just a portion of the whole circulatory system and since the 3D modelling will be limited to the heart and to the initial tracts of the main vessels, boundary conditions must be applied at the inlets and outlets of the model, so to account for the resistive, elastic and inertial features of the missing vascular network. These features are generally represented into a lumped parameter network whose description requires inexpensive differential equations (analogous to those of electrical circuits)^56^. As in the present cardiac model the inlets/outlets are embedded in the computational domain, the boundary conditions are imposed through the volume forcing ${\textbf{f}}_{WK}$ in Eq. (1), which is only active in the cylindrical subdomains (having outward-pointing normal vector $\textbf{n}_\Omega$) indicated in Fig. 8. The forcing is given by $-\textbf{f}_{WK}=\alpha {\textbf{u}} +\beta \int _0^t {\textbf{u}}(\tau ) \textrm{d} \tau + \gamma {\textbf{n}}_\Omega$, which along with the resistance and capacitance of the initial tracts of the veins/arteries is equivalent to a three elements *Windkessel*^57,58^ (see Table 3). This open-loop approach where each inflow/outflow boundary conditions is provided separately could be improved by resorting to closed-loop models where each outflow condition is coupled through a system of differential equations to the corresponding inlet condition^59–61^, e.g. the outflow of the descending aorta to the inlet of the inferior vena cava with the 0D model mimicking the lower-body systemic circulation.

**Table 3:** Windkessel parameters at the inlets/outlets of the cardiac model as defined in Fig. 8.

	$\Omega _{ao}$	$\Omega _{epi}$	$\Omega _{pv}$	$\Omega _{pa}$	$\Omega _{vcs}$	$\Omega _{vci}$
$\alpha ~\text {(Kg } \text {m }^{-3} \text { s }^{-1} \times 10^6)$	3.13	16.62	0.062	0.78	0.39	0.39
$\beta ~\text {(Kg }\text { m }^{-3} \text { s }^{-2} \times 10^6)$	2.96	10.36	0.059	1.18	0.11	0.11
$\gamma ~\text {(Kg }\text { m }^{-2} \text { s }^{-2} \times 10^6)$	18.43	25.68	0.00	4.17	0.00	0.00

#### Structural solver

The dynamics of the deformable heart tissues is solved using a spring-network structural model based on the Fedosov’s interaction potential approach^62^. A 3D solver is used for the ventricular and atrial myocardium that are discretized using a tetrahedral mesh, with the endocardium wet by the blood corresponding to a triangular inner surface. On the other hand, thin membranes as the valve leaflets are discretized through 2D triangulated surfaces. Several models of the elasticity of the myocardium are available in the literature, also accounting for its orthotropic properties^45,63,64^. Here, the orthotropic and hyperelastic nature of biological cardiac tissues is modelled by a larger elastic stiffness in the fiber direction, $\hat{\textbf{e}}_\parallel$, than in the sheet, $\hat{\textbf{e}}_/$, and sheet-normal, $\hat{\textbf{e}}_\perp$, directions and by a nonlinear strain–stress behaviour according to a Fung-type constitutive relation, where the strain energy density reads $W_e = \frac{c}{2}(e^Q-1),$ with $Q=\alpha _\parallel \epsilon _{\parallel }^2+\alpha _/ \epsilon _{/}^2 +\alpha _\perp \epsilon _{\perp }^2$ being a combination of the Green strain tensor components in the fiber, $\epsilon _{\parallel }$, sheet, $\epsilon _{/}$, and sheet-normal $\epsilon _{\perp }$ directions. The coefficients *c*, $\alpha _\parallel$, $\alpha _/$, $\alpha _\perp$ have been set as in Table 4 so as to reproduce the stress-strain curves in the fiber and cross-fiber direction measured ex-vivo in different portions of the cardiac tissue.

**Table 4:** Elastic parameters of the Fung constitutive relation for the various cardiac components.

Cardiac tissue	*C* (KPa)	$\alpha _{\parallel }$	$\alpha _{/}=\alpha _{\perp }$	References
Left ventricle	11.59	9.97	3.17	Calibrated on biaxial tests of ovine cardiac tissue^65^
Left atrium	8.31	3.77	3.52	Calibrated on biaxial tests of ovine cardiac tissue^65^
Right ventricle	9.20	8.22	4.09	Calibrated on biaxial tests of ovine cardiac tissue^65^
Right atrium	2.95	6.50	6.52	Calibrated on biaxial tests of ovine cardiac tissue^65^
Arteries and veins	16.77	16.39	15.76	Calibrated on biaxial tests of aortic human tissue^66^
Mitral and tricuspid valve	0.14	98.58	71.24	Calibrated on biaxial tests of porince mitral leaflet^67^
Aortic and pulmonary valve	0.0093	120.88	5.87	Calibrated on biaxial tests of porcine aortic leaflet^68^

#### Electrophysiology solver

The heterogeneous properties of the electrophysiology network are captured by resorting to a state-of-the-art electrical model of the whole heart^31^. Specifically, the cardiac geometry is decomposed into a set of coupled conductive media having different topology and electrical conductivities: (i) a network of slender *bundles* comprising a fast conduction atrial network, the *AV–node* and the ventricular bundles; (ii) the *Purkinje network*; and (iii) the atrial and ventricular myocardium (see Fig. 1e). The propagation of the cellular *action potential* in (iii) is governed by the bidomain equations:

$$\begin{aligned}{} & {} \chi \left( C_m \frac{\partial v}{\partial t} +I_{ion}(\textbf{s}) + I_s \right) = \nabla \cdot (\mathscr {M}^{int} \nabla v) + \nabla \cdot (\mathscr {M}^{int} \nabla v_{ext}),\\{} & {} \quad 0 = \nabla \cdot (\mathscr {M}^{int} \nabla v + (\mathscr {M}^{int}+ \mathscr {M}^{ext}) \nabla v_{ext}),\\{} & {} \quad \frac{ \textrm{d} \textbf{s}}{ \textrm{d} t} = F(\textbf{s},v,t), \end{aligned}$$

where *v* and $v_{ext}$ are the transmembrane and extracellular potential, $\chi$ and $C_m$ are the surface-to-volume ratio of cells and the membrane capacitance and $I_s$ is the external triggering stimulus initiating the myocardial depolarization placed in the sino-atrial node. The intracellular, $\mathscr {M}^{int}$, and extracellular, $\mathscr {M}^{ext}$, conductivity tensors are set to reflect the orthotropic myocardium electrical properties and thus depend both on the conductive media and on the local fiber orientation (Fig. 1c,d). In the case of a 3D conductive media, as the myocardium, these tensors have rank three and are diagonal when expressed in the fiber $(\parallel )$, sheet-fiber (/) and cross-fiber $(\perp )$ directions, having components $m^{ext}_\parallel$, $m^{ext}_/$, $m^{ext}_\perp$ and $m^{int}_\parallel$, $m^{int}_/$, $m^{int}_\perp$. Since for the fast conductive bundles and the Purkinje network the external and the internal conductivity tensors can be taken as proportional one to the other^31^ $\mathscr {M}^{ext}=\lambda \mathscr {M}^{int}$, the bidomain system of PDEs reduces to the monodomain equation $\chi \left( C_m \frac{\partial v}{\partial t} +I_{ion}(\textbf{s}) + I_s \right) = \nabla \cdot (\mathscr {M} \nabla v)$, with $\mathscr {M}= \lambda \mathscr {M}^{int}/(1+\lambda )$. The values of the principal conductivities components over the cardiac domain are reported in Table 5. The set of bidomain/modomain equations is solved using an in-house finite volume library, which provides a suitable approach for solving the electrophysiology equations in complex geometries^31^, and it is coupled through the ionic current per unit cell membrane $I_{ion}$ to three different *cellular models* (indicated by the last equation): the Courtemanche model^69^ for the atrial myocytes, the Stewart model^70^ for the Purkinje network and the ten Tusscher-Panfilov model^71^ for the ventricular myocytes. The active muscular tension ${\textbf{ F}}_n^{act}$ at the mesh cell is then obtained as a function of the transmembrane potential *v* through the model equation proposed by Nash and Panfilov ^72^.

**Table 5:** Electrical conductivities and electrophysiology/cell models of the various cardiac components.

Cardiac tissue	PDE model	Cell model	Conductivity values mS/mm	References
Left and right ventricles	Bidomain	ten Tusscher-Panfilov^71^	$$m^{ext}_\parallel =0.62$$, $$m^{ext}_/=m^{ext}_\perp =0.24$$$$m^{int}_\parallel =0.17$$, $$m^{int}_/=m^{int}_\perp =0.019$$	^3^
Left and right atria	Bidomain	Courtemanche^69^	$$m^{ext}_\parallel =0.66$$, $$m^{ext}_/=m^{ext}_\perp =0.25$$$$m^{int}_\parallel =0.18$$, $$m^{int}_/=m^{int}_\perp =0.02$$	Calibrated for a longitudinal speed of 0.5 m/s^73,74^
Purkinje network	Monodomain	Stewart^70^	$m_\parallel =m_/=3.95$	Calibrated for a depolarization speed of 4.0 m/s^73^
Internodal bundles	Monodomain	Courtemanche^69^	$m_\parallel =1.29$	Calibrated for a depolarization speed of 1.54 m/s^74^

#### Coupling

The contraction and relaxation of the heart chambers along with the passive motion of the vessels and valve leaflets result from the dynamic balance among the inertia of the tissues, the external hydrodynamic forces given by the fluid solver ${\textbf{ F}}_n^{ext}$, the internal passive forces coming from the structural solver ${\textbf{ F}}_n^{int}$ and the active tension computed by the electrophysiology solver ${\textbf{ F}}_n^{act}$: $~~~m_n\frac{ \textrm{d}^2 {\textbf{x}}_n}{\textrm{d} t^2} = {\textbf{ F}}_n^{ext} + {\textbf{ F}}_n^{int} + {\textbf{ F}}_n^{act},~~~$ where $m_n$ is the tissue mass associated with the $n^{th}-$Lagrangian mesh node and ${\textbf{x}}_n$ its (instantaneous) position. The hydrodynamics force is non-zero only on the mesh nodes belonging to the wet surfaces (namely the valve leaflets and the inner wall of the heart chambers/vessels), whereas the active tension can be non-zero only for the nodes belonging to the muscular myocardium, i.e. ventricles and atria. Both, strong and loose coupling approaches have been implemented in the code^28,30^ . The first is based on a predictor-corrector two-step Adams-Bashforth scheme and the three solvers–fluid, structure and electrophysiology—are iterated (typically 2–3 times) until the maximum relative error computed on the position and velocity of the structural nodes decreases below a prescribed threshold (usually $10^{-4}$). In the loose coupling method, fluid and electrophysiology are solved first and the generated hydrodynamic and active loads are used to evolve the structure, whose updated configuration is the input for the successive time step. This approach is computationally cheaper than the strong coupling but is proner to numerical instability thus a smaller time step has to be used to integrate the equations. The small time step used here ($\Delta t =2\mu s$) ensures the stability of the loose coupling procedure.

### Synthetic ECG

The heart model has been enclosed in the idealized torso shown in Fig. 1a, which has been constructed to represent an average patient geometry having a heart-to-skin distance of 35 mm, in line with the parasternal average value of 32.1±7.9 mm (measured on a total of 150 individuals, 71 male and 79 female^75^). The waist circumference of 92.5 cm is within the normal range for the female and male populations (defined as mean value ± twice the standard deviation^76^), which are respectively equal to 66.1–106.1 cm (mean 86.1 cm, measured on 1986 subjects) and 71.7–116.4 cm (mean 94.1 cm, measured on 4082 subjects). The chest circumference is equal to 107.3 cm, which is also within the normal ranges of the female (78.2–111.2 cm; mean 94.7 cm) and male (88.4–123.4 cm; mean 105.9 cm) populations measured on the same groups^76^. In Fig. 1a are also indicated the surface locations used to calculate the ECG. The voltage difference between these two leads examines the cardiac depolarization along the junction between atria and ventricles (heart vertical axis), with negative electrical potentials corresponding to electrical wavefronts moving towards the apex of the heart. The surface potential at the ECG leads, $V_s$, can be obtained by solving the electrical potential within the torso coupled with the cardiac electrophysiology system^77^. Alternatively, in the assumption of isotropic electrical conductivity in the torso, $V_s$ at a surface position ${\textbf{x}}_s$ and time *t* is given by^78^:

$$\begin{aligned} V_s({\textbf{x}}_s,t) = - K \int _{\Omega _{heart}} \nabla v(\textbf{x},t) \cdot \nabla \left( \frac{1}{|| {\textbf{x}} - {\textbf{x}}_s ||}\right) \textrm{d} \textbf{x}, \end{aligned}$$

where $\Omega _{heart}$ indicates the cardiac domain where the electrophysiology bidomain equations are solved, $\nabla v({\textbf{x}})$ is the spatial gradient of the transmembrane potential at the cardiac location ${\textbf{x}}$ and *K* includes the ratio between the intracellular and torso conductivity.

### GPU acceleration

A drawback of the FSEI is that it requires a large computational power implying long time to obtain results. GPUs, however, have emerged as a convenient platform for high performance computing as they allow for unprecedented speed-ups and, consequently, considerable reductions of the time-to-solution. To this aim, the code has been ported to CUDA-Fortran^30^ and the GPU-accelerated FSEI algorithm can now tackle complex cardiac simulations with $\sim$ one billion of spatial degrees (including the demanding solution of the Navier-Stokes equations) within a few hours, thus allowing for running in-silico clinical trials.

## Supplementary Information

Supplementary Information.

## Data Availability

The datasets used and/or analyzed during the current study are available from the corresponding author on reasonable request.
