# Supplementary material for: GPU accelerated digital twins of the human heart open new routes for cardiovascular research
Source: Sci Rep. 2023 May 22;13:8230. doi: 10.1038/s41598-023-34098-8 (PMC10203142; doi:10.1038/s41598-023-34098-8)
Supplement: Supplementary file 1 — Supplementary Information. [file 41598_2023_34098_MOESM1_ESM.pdf]

GPU accelerated digital twins of the human heart open new routes for cardiovascular research – *Supplementary Material*

Francesco Viola[1], **Giulio Del Corso**[1], **Ruggero De Paulis**[2,3], **and Roberto Verzicco**[1,4,5,*]

[1]Gran Sasso Science Institute (GSSI), L'Aquila, Italy.
[2]European Hospital of Rome, Italy.
[3]Unicamillus, Rome, Italy.
[4]University of Rome Tor Vergata, Rome, Italy.
[5]POF Group, University of Twente, Enschede, The Netherlands.
[*]roberto.verzicco@gssi.it

ABSTRACT

The recruitment of patients for rare or complex cardiovascular diseases is a bottleneck for clinical trials and digital twins of the human heart have recently been proposed as a viable alternative.
In this paper we present an unprecedented cardiovascular computer model which, relying on the latest GPU–acceleration technologies, replicates the full cardiac dynamics within a few hours. This opens the way to extensive simulation campaigns to study the response of synthetic cohorts of patients to cardiovascular disorders, novel prosthetic devices or surgical procedures. As a proof–of–concept we show the results obtained for left bundle branch block disorder and the subsequent cardiac resynchronization obtained by pacemaker implantation. The in–silico results closely match those obtained in clinical practice, confirming the reliability of the method.
This innovative approach makes possible a systematic use of digital twins in cardiovascular research, thus reducing the need of real patients with their economical and ethical implications. This study is a major step towards in–silico clinical trials in the era of digital medicine.

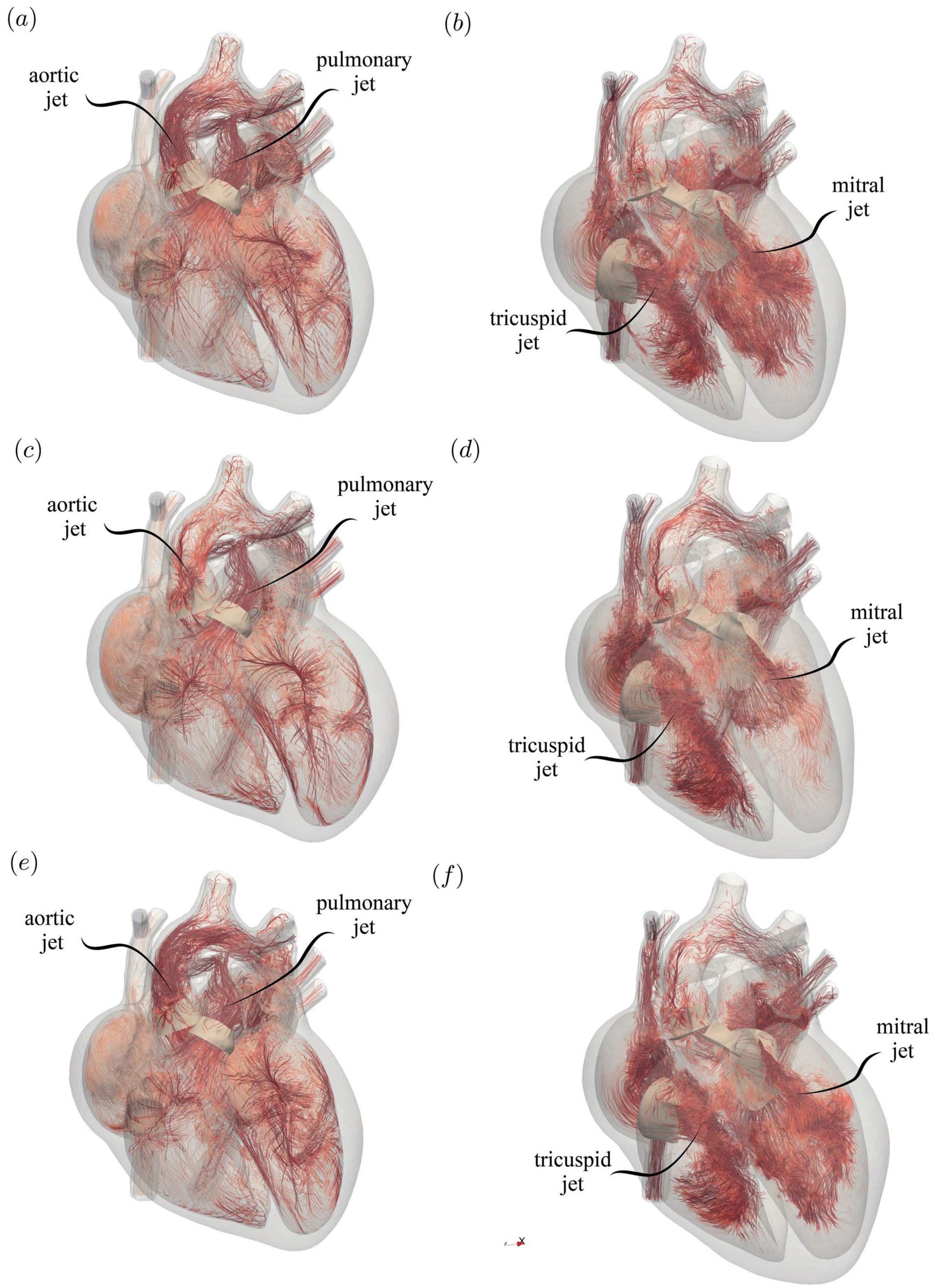

Figure 1. Three–dimensional cardiac hemodynamics. Instantaneous snapshot of the flow streamlines coloured with the velocity magnitude (0 m/s white, 1.5 m/s dark red). **a,** Systole and **b,** diastole for the case of healthy patient. **c,** Systole and **d,** diastole for the pathologic case with left bundle branch block (LBBB). **e,** Systole and **f,** diastole for the resynchronized case with the left ventricular lead in the optimal position LP1 (LBBB+CRT/LP1).

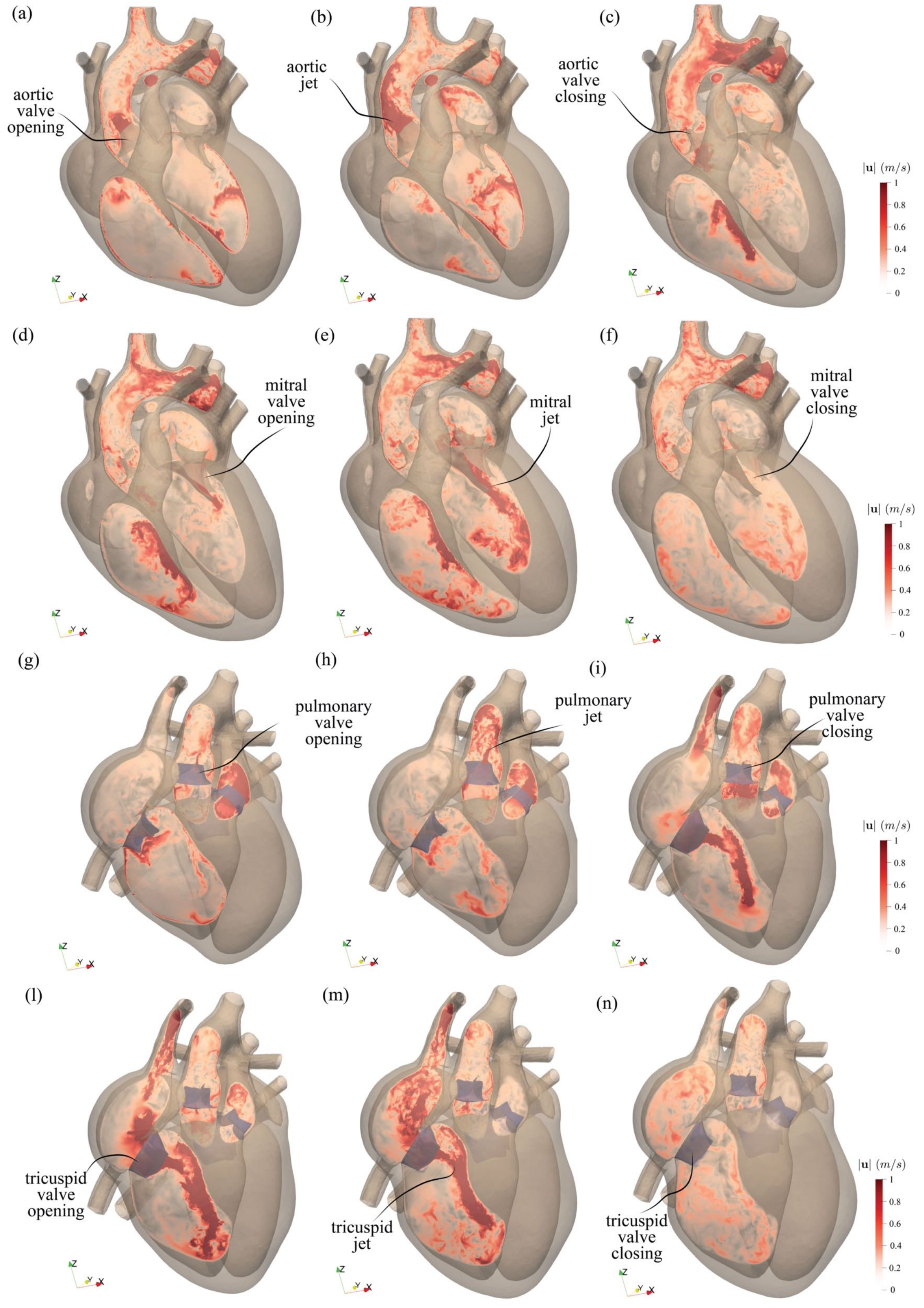

Figure 2. Cardiac hemodynamics. Instantaneous snapshots of the blood velocity magnitude over plane sections crossing the left (**a,–f,**) and right (**g,–l,**) parts of the heart for the pathologic case with left bundle branch block (LBBB). The left plane position is such to cross in the middle the mitral and aortic valves. Similarly, the right plane crosses the pulmonary and tricuspid valve. **a,** and **g,** $t = 520$ms, **b,** and **h,** $t = 560$ms, **c,** and **i,** $t = 620$ms, **d,** and **j,** $t = 640$ms, **e,** and **k,** $t = 700$ms, **f,** and **l,** $t = 800$ms.

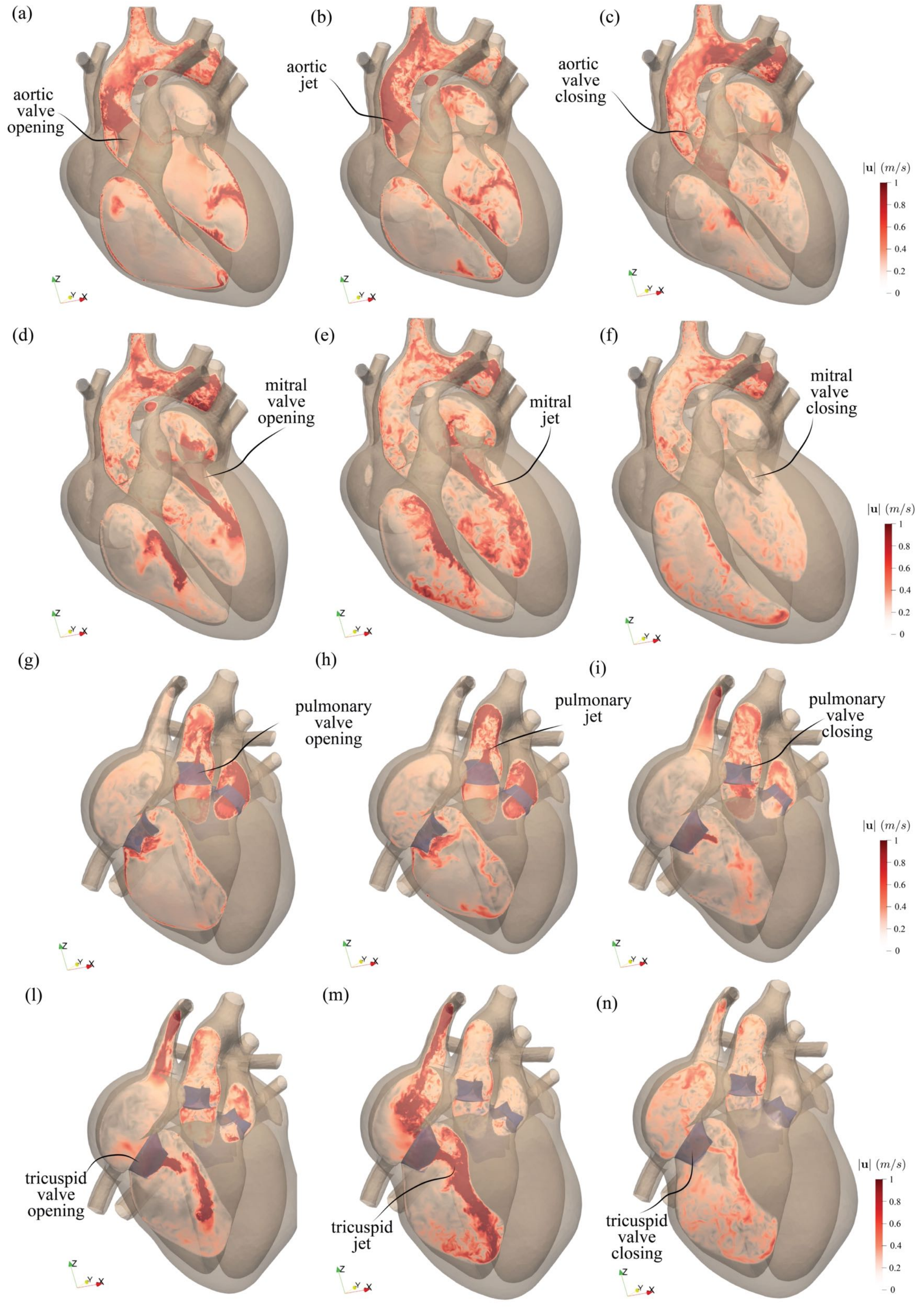

Figure 3. Cardiac hemodynamics. Instantaneous snapshots of the blood velocity magnitude over plane sections crossing the left (**a,–f,**) and right (**g,–l,**) parts of the heart for the resynchronized case with the left ventricular lead in the optimal position LP1 (LBBB+CRT/LP1). The left plane position is such to cross in the middle the mitral and aortic valves. Similarly, the right plane crosses the pulmonary and tricuspid valve. **a,** and **g,** $t = 500$ms, **b,** and **h,** $t = 540$ms, **c,** and **i,** $t = 600$ms, **d,** and **j,** $t = 620$ms, **e,** and **k,** $t = 680$ms, **f,** and **l,** $t = 780$ms.

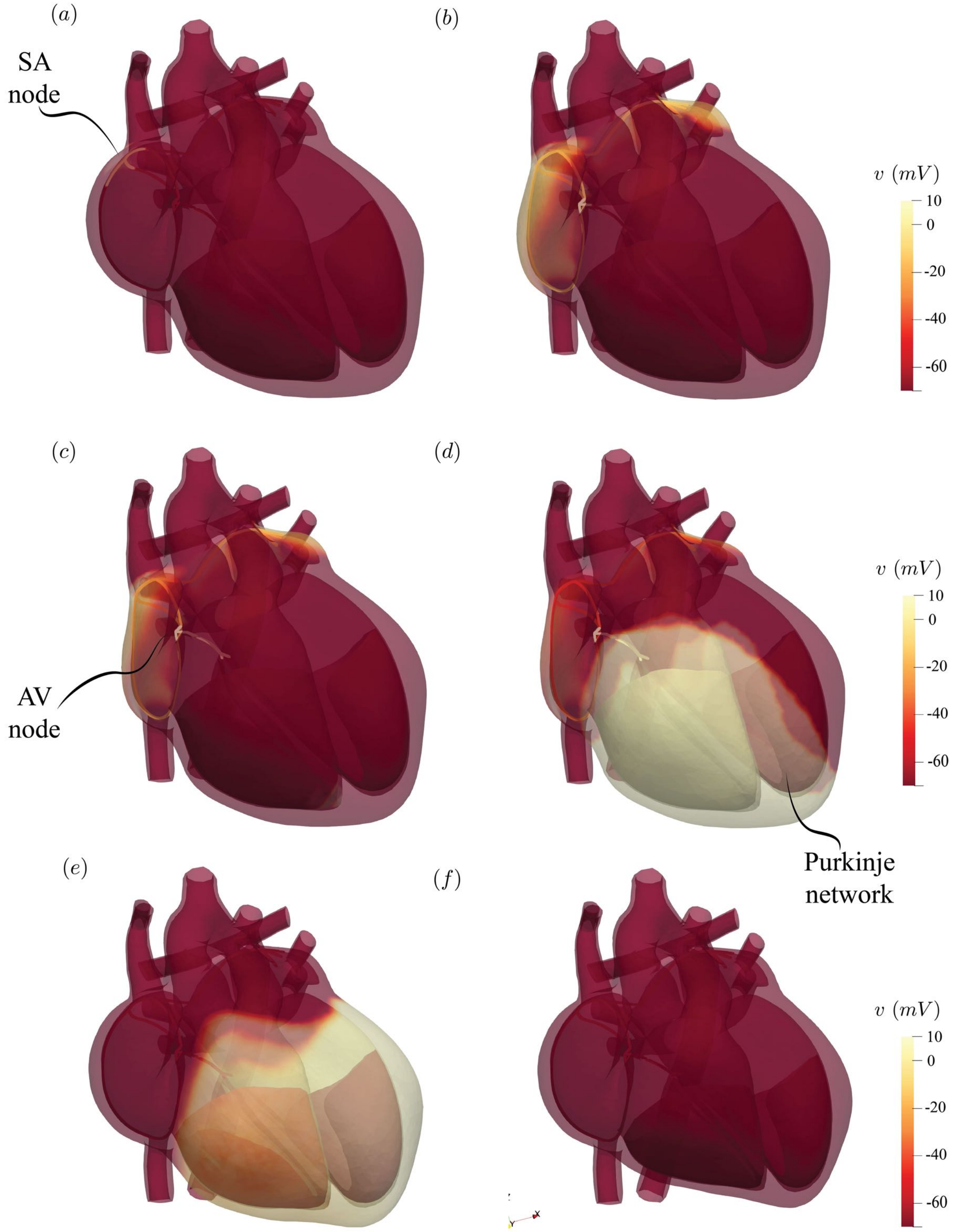

Figure 4. Depolarization of the electrophysiology network. Instantaneous snapshots of the activation potential during the heartbeat for the pathologic case with left bundle branch block (LBBB). The sequence of the snapshots is the same as Figure 2 of the manuscript.

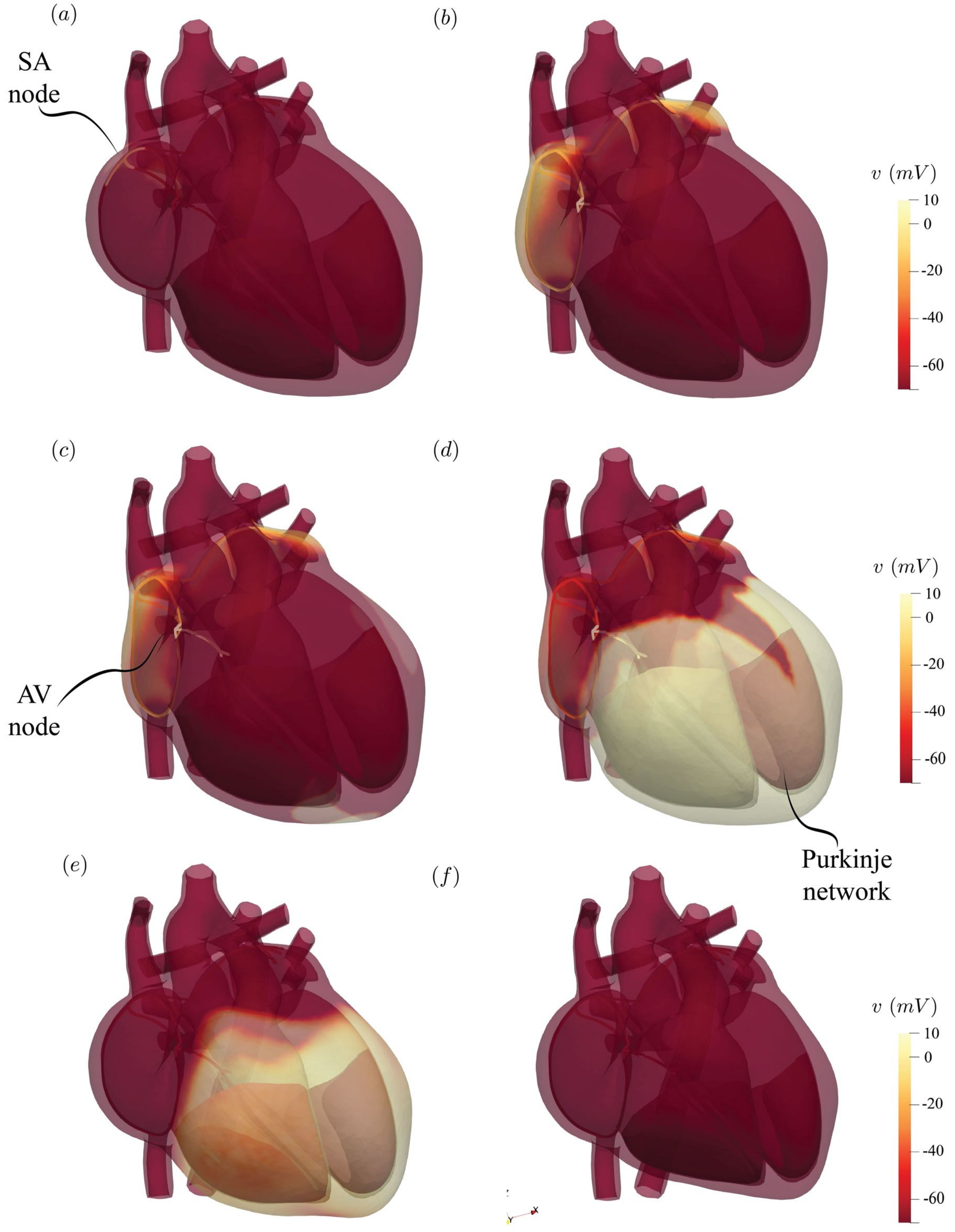

Figure 5. Depolarization of the electrophysiology network. Instantaneous snapshots of the activation potential during the heartbeat for the resynchronized case with the left ventricular lead in the optimal position LP1 (LBBB+CRT/LP1). The sequence of the snapshots is the same as Figure 2 of the manuscript.

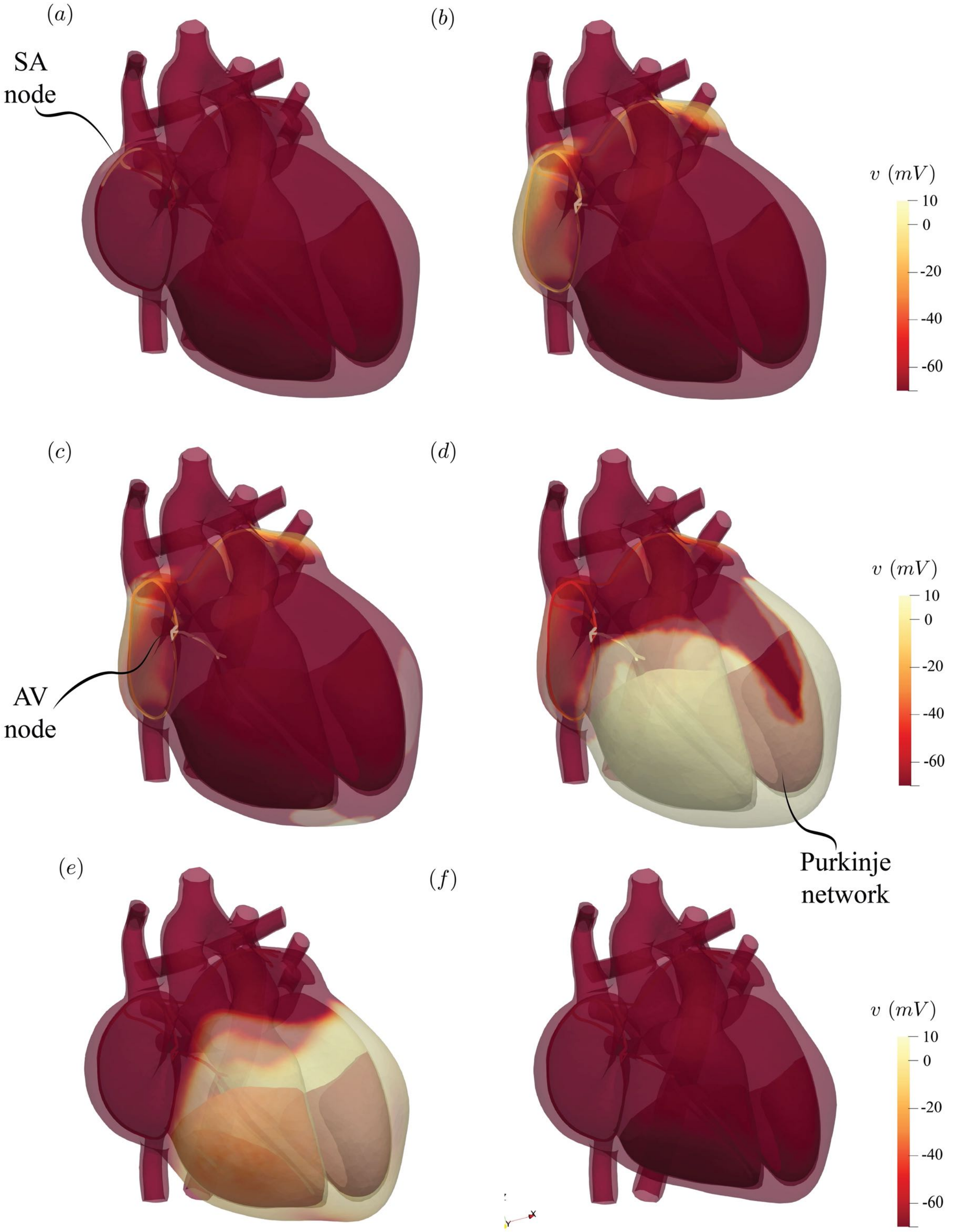

Figure 6. Depolarization of the electrophysiology network. Instantaneous snapshots of the activation potential during the heartbeat for the resynchronized case with the left ventricular lead in the position LP2 (LBBB+CRT/LP2). The sequence of the snapshots is the same as Figure 2 of the manuscript.

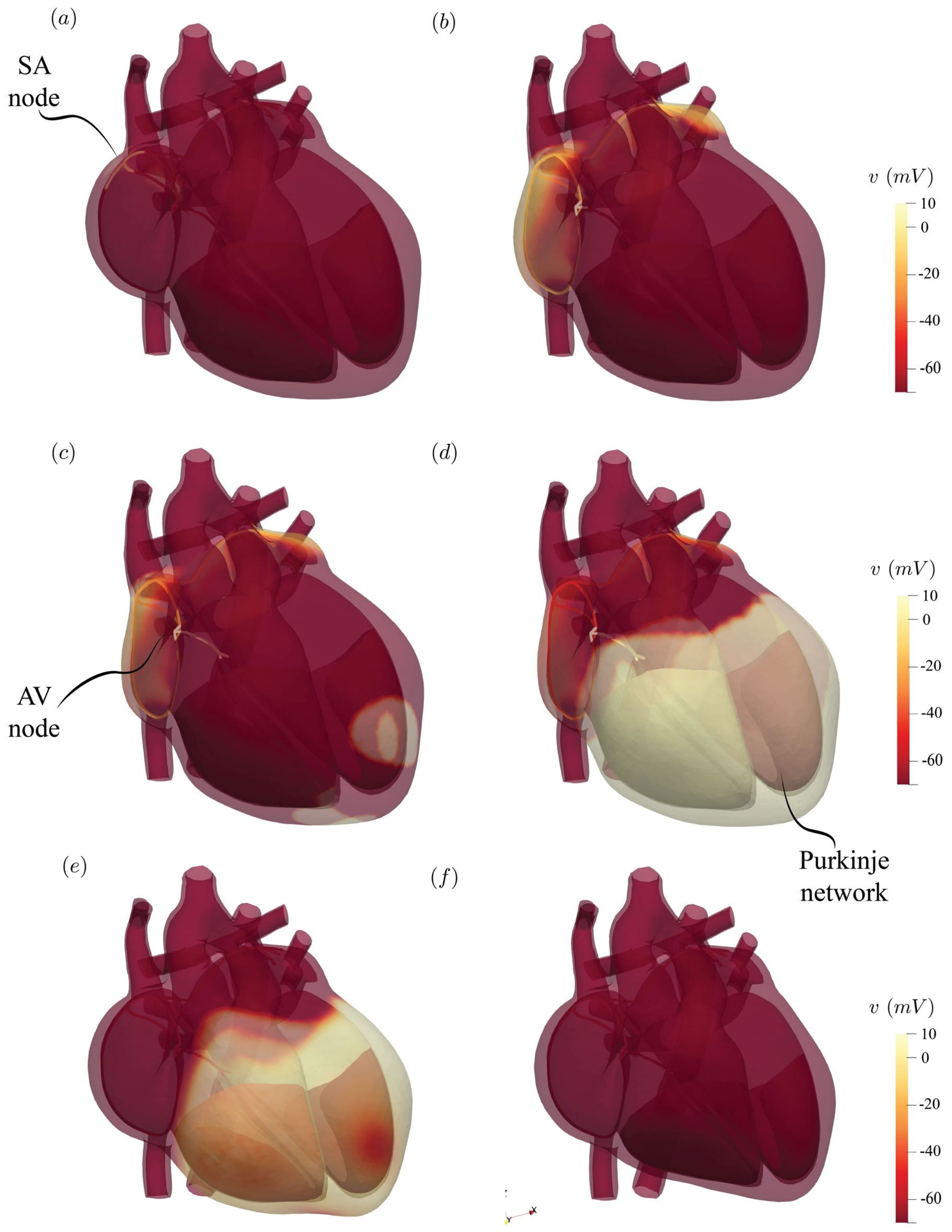

Figure 7. Depolarization of the electrophysiology network. Instantaneous snapshots of the activation potential during the heartbeat for the resynchronized case with the left ventricular lead in the position LP3 (LBBB+CRT/LP3). The sequence of the snapshots is the same as Figure 2 of the manuscript.

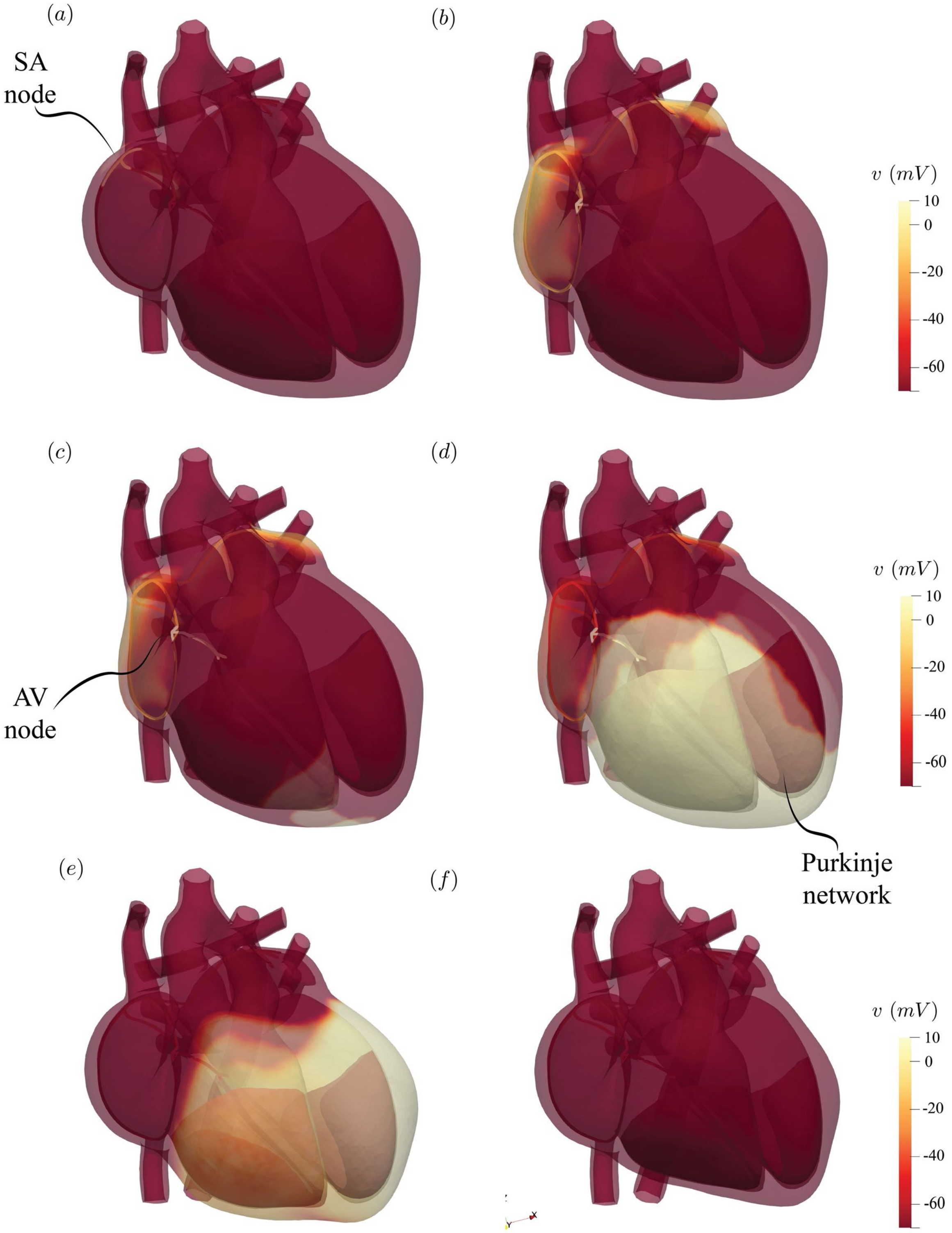

Figure 8. Depolarization of the electrophysiology network. Instantaneous snapshots of the activation potential during the heartbeat for the resynchronized case with the left ventricular lead in the position LP4 (LBBB+CRT/LP4). The sequence of the snapshots is the same as Figure 2 of the manuscript.

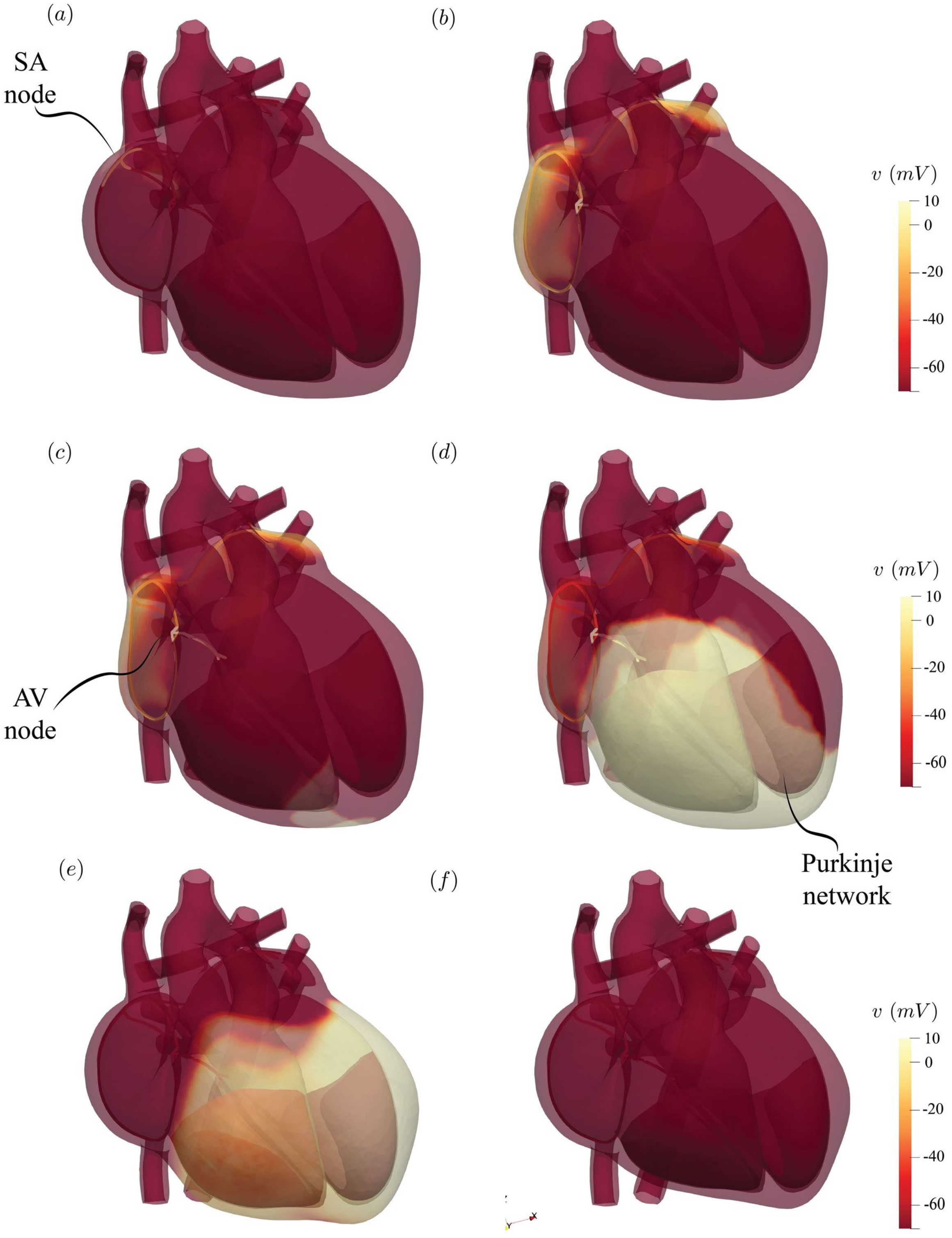

Figure 9. Depolarization of the electrophysiology network. Instantaneous snapshots of the activation potential during the heartbeat for the resynchronized case with the left ventricular lead in the position LP5 (LBBB+CRT/LP5). The sequence of the snapshots is the same as Figure 2 of the manuscript.

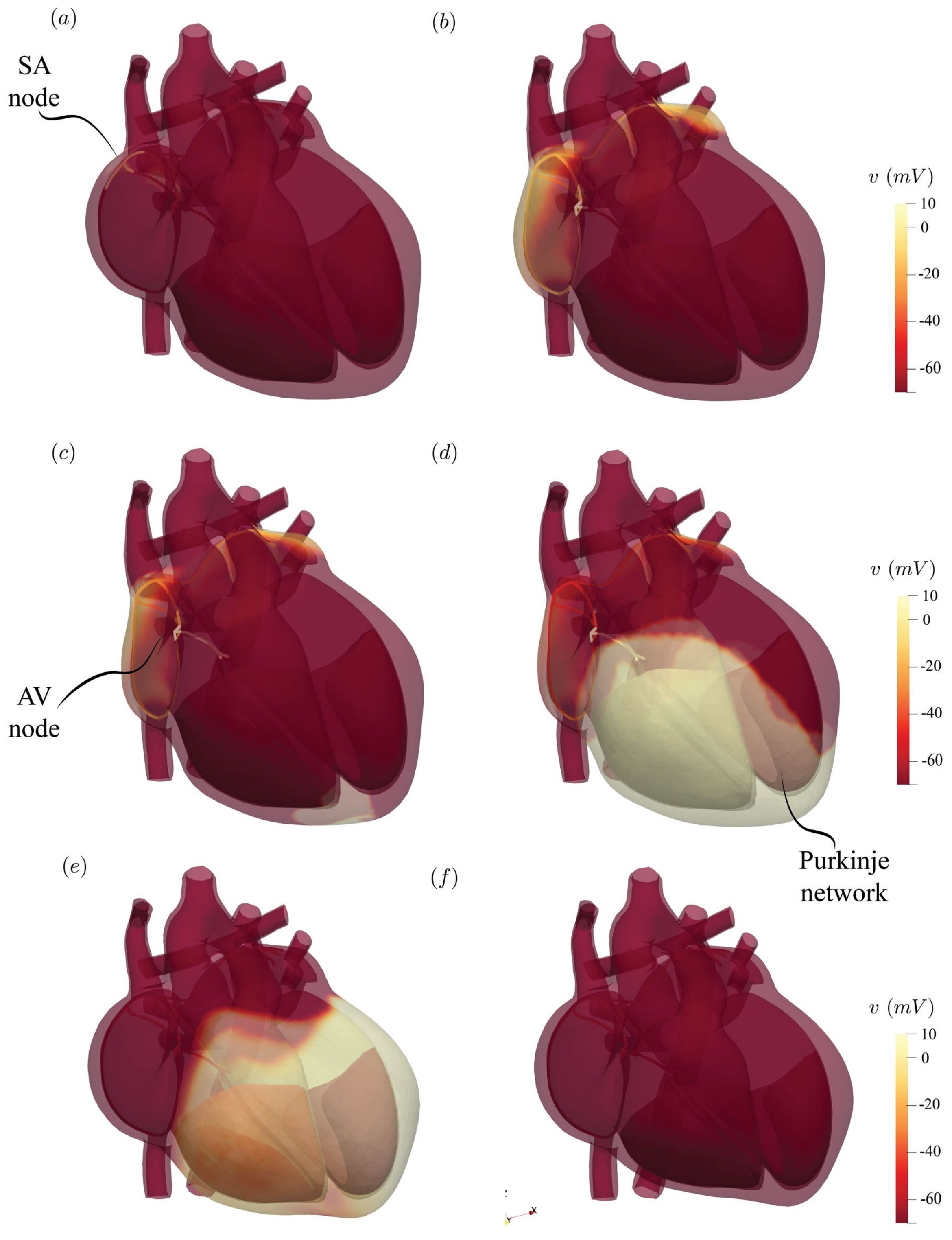

Figure 10. Depolarization of the electrophysiology network. Instantaneous snapshots of the activation potential during the heartbeat for the resynchronized case with the left ventricular lead in the position LP6 (LBBB+CRT/LP6). The sequence of the snapshots is the same as Figure 2 of the manuscript.

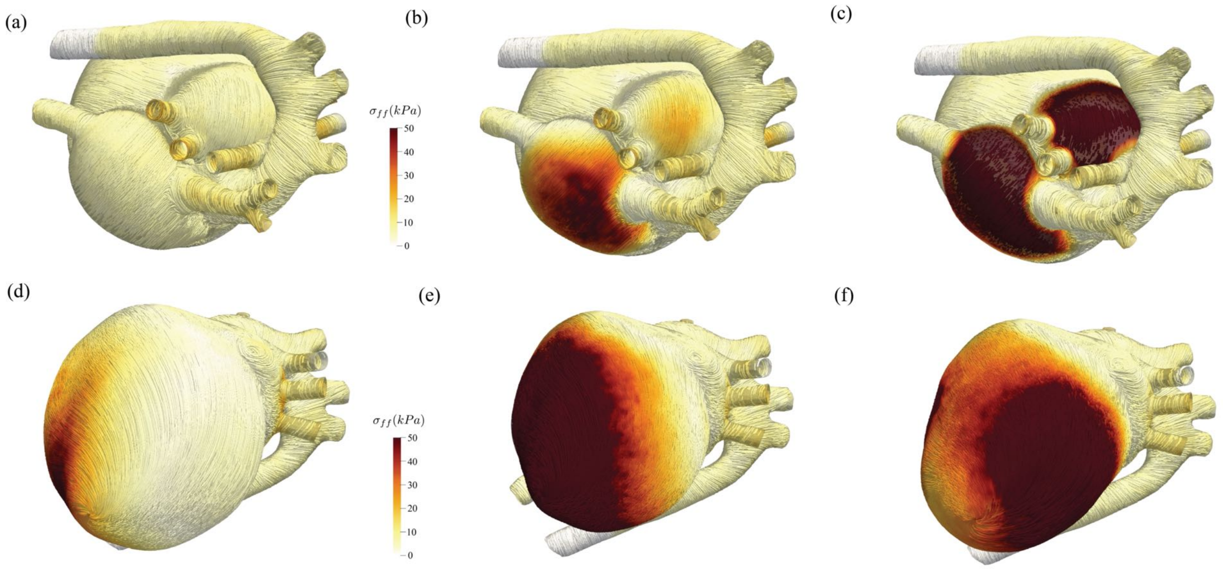

Figure 11. Tissue stresses. Instantaneous surface distribution of the tension along the fibres axes (force per unit area) during a heartbeat for the pathologic case with left bundle branch block (LBBB). The sequence of the snapshots is the same as Figure 4 of the manuscript.

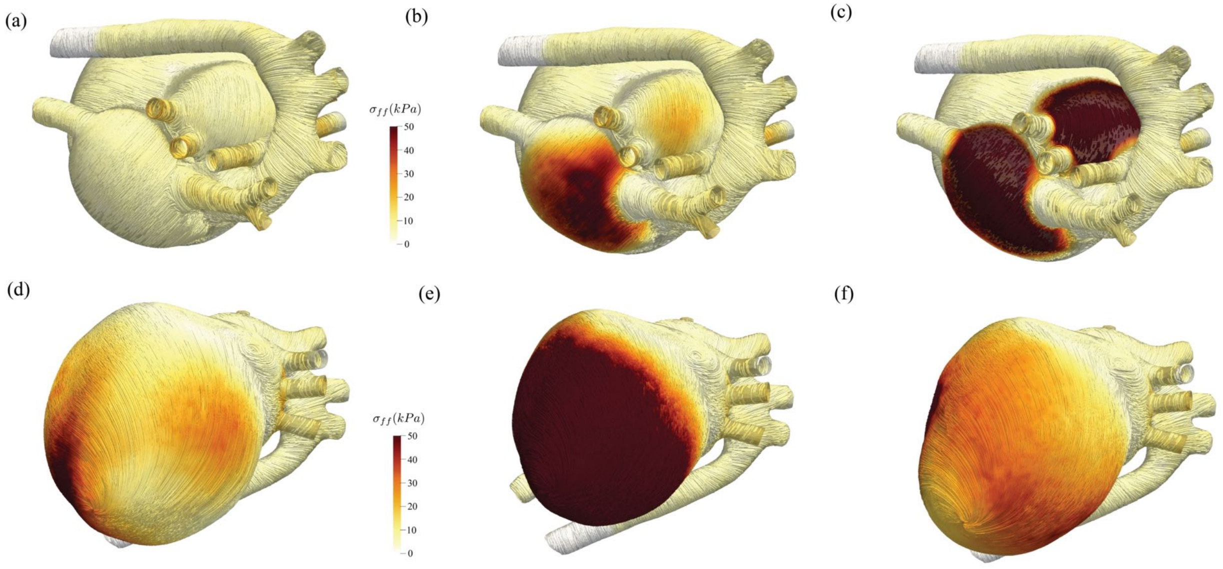

Instantaneous surface distribution of the tension along the fibres axes (force per unit area) during a heartbeat

Figure 12. Tissue stresses. Instantaneous surface distribution of the tension along the fibres axes (force per unit area) during a heartbeat for the resynchronized case with the left ventricular lead in the optimal position LP1 (LBBB+CRT/LP1). The sequence of the snapshots is the same as Figure 4 of the manuscript.

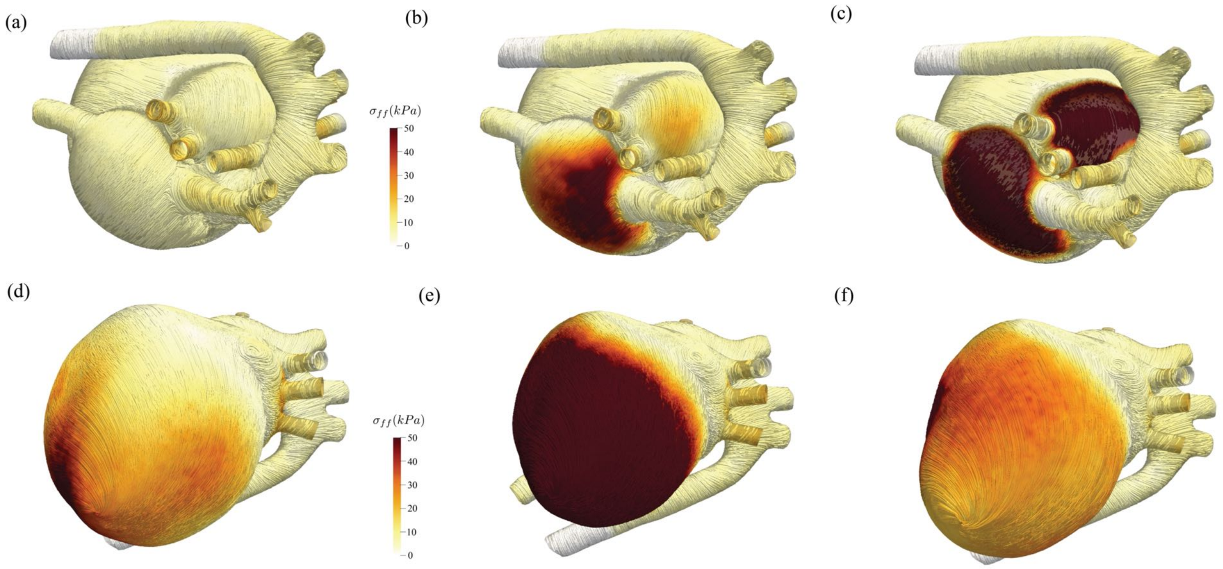

Figure 13. Tissue stresses. Instantaneous surface distribution of the tension along the fibres axes (force per unit area) during a heartbeat for the resynchronized case with the left ventricular lead in the position LP2 (LBBB+CRT/LP2). The sequence of the snapshots is the same as Figure 4 of the manuscript.

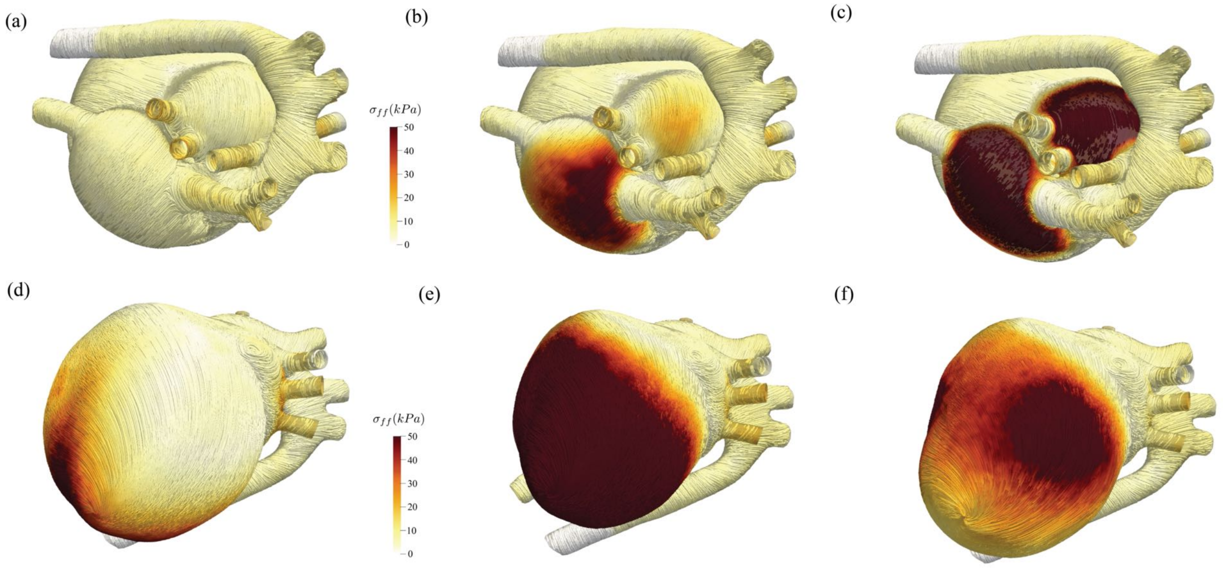

Figure 14. Tissue stresses. Instantaneous surface distribution of the tension along the fibres axes (force per unit area) during a heartbeat for the resynchronized case with the left ventricular lead in the position LP3 (LBBB+CRT/LP3). The sequence of the snapshots is the same as Figure 4 of the manuscript.

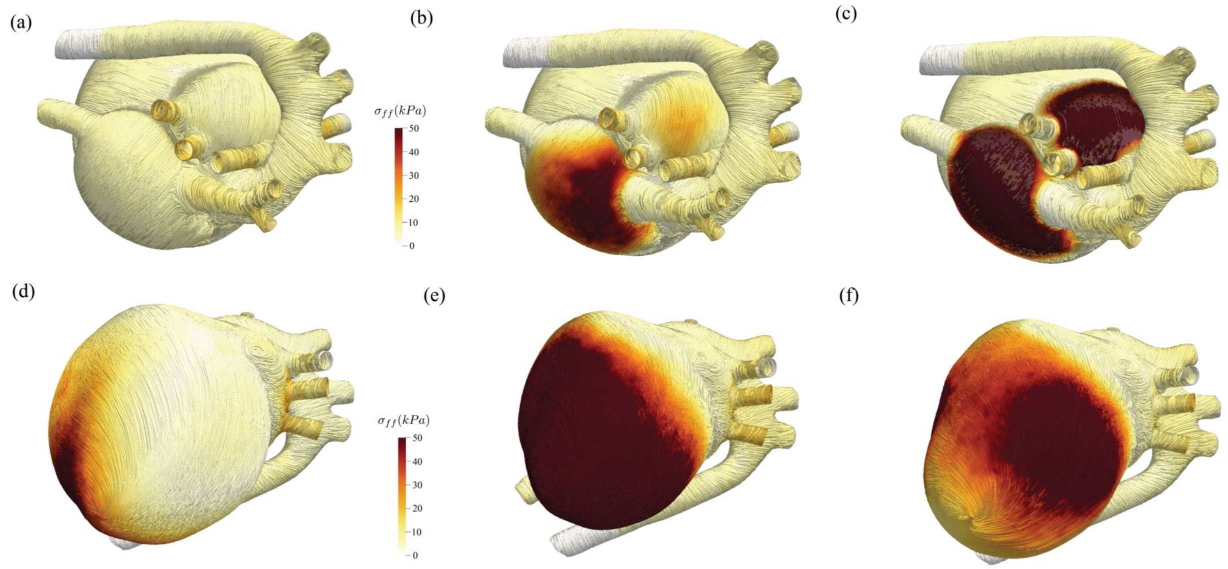

Figure 15. **Tissue stresses.** Instantaneous surface distribution of the tension along the fibres axes (force per unit area) during a heartbeat for the resynchronized case with the left ventricular lead in the position LP4 (LBBB+CRT/LP4). The sequence of the snapshots is the same as Figure 4 of the manuscript.

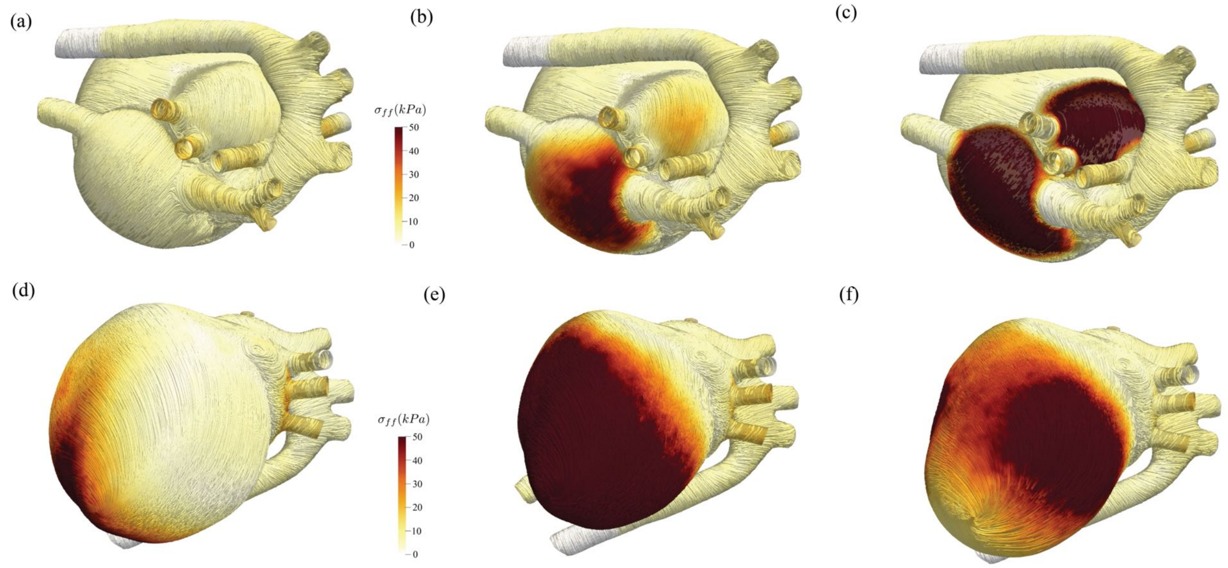

Figure 16. **Tissue stresses.** Instantaneous surface distribution of the tension along the fibres axes (force per unit area) during a heartbeat for the resynchronized case with the left ventricular lead in the position LP5 (LBBB+CRT/LP5). The sequence of the snapshots is the same as Figure 4 of the manuscript.

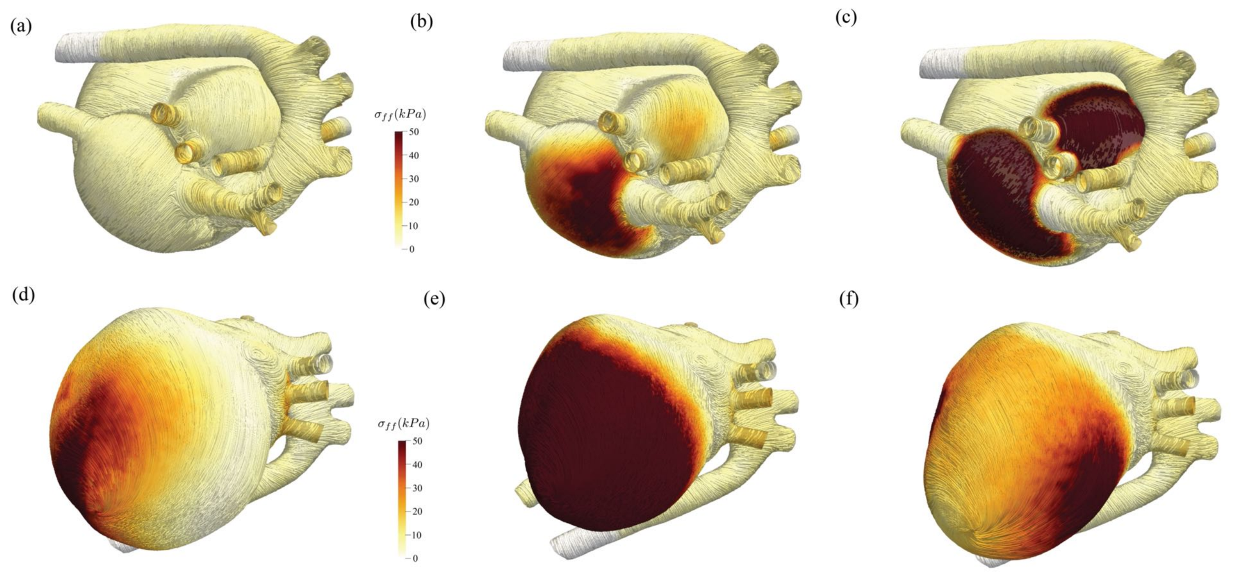

Figure 17. Tissue stresses. Instantaneous surface distribution of the tension along the fibres axes (force per unit area) during a heartbeat for the resynchronized case with the left ventricular lead in the position LP6 (LBBB+CRT/LP6). The sequence of the snapshots is the same as Figure 4 of the manuscript.
